# Neuroprotective effect of Mulmina Mango against chemotherapy-induced cognitive decline in mouse model of mammary carcinoma

**DOI:** 10.1038/s41598-022-06862-9

**Published:** 2022-02-23

**Authors:** Jeena John, Manas Kinra, Niraja Ranadive, Raghuvir Keni, Pawan Ganesh Nayak, Rajesh N. Jagdale, Syed M. Ahmed, Kaggundi V. Raghavendra, Jayesh Mudgal, K. Nandakumar

**Affiliations:** 1 Department of Pharmacology, Manipal College of Pharmaceutical Sciences, Manipal Academy of Higher Education, Manipal, Karnataka 576104 India; 2 Juggat Pharma, Jagdale Industries Pvt. Ltd., Bengaluru, Karnataka 560078 India

**Keywords:** Cancer, Neuroscience, Biomarkers

## Abstract

The post-treatment status of breast cancer survivors has become a concern because of the toxicity induced by chemotherapeutic agents in the brain tissues resulting in cognitive deficits, which is generally referred as chemobrain. The aim of this study was to assess the effect of a proprietary ayurvedic formulation Mulmina Mango against chemotherapy-induced cognitive impairment (CICI). Mammary carcinoma was induced by subcutaneously inoculating 4T1 cells into the mammary fat pad of the animals. Intraperitoneal administration of Cyclophosphamide, Methotrexate, 5-Fluorouracil (CMF) regimen was carried out once a week for three weeks. Treatment of Mulmina began one week before chemotherapy and continued till the end of the chemotherapy cycle. After three cycles of chemotherapy, cognitive decline was assessed by Morris water maze task followed by assessment of locomotor activity by open-field test. Tumor progression was evaluated by measurement of tumor volume. Oxidative and neuroinflammatory markers were also evaluated from the isolated brain samples. CMF treatment resulted in a considerable reduction in tumour volume. We found chemotherapy negatively affected behavioral and biochemical parameters in animals and Mulmina treatment ameliorated these cognitive impairments by restoring antioxidant and maintaining cytokine levels. The combination of phytochemicals in Mulmina proved its possible ability to alleviate CICI without affecting chemotherapeutic efficiency and could pave the way for identifying treatment strategies to combat chemobrain.

## Introduction

Breast cancer in women is one of the most common types of cancers, with approximately 281,550 new cases expected in the United States in 2021. But the early detection and improved treatment therapies significantly reduced mortality rates and increased survival rates. According to the American Cancer Association, survival rates for women diagnosed with breast cancer are 91% at five years, 84% at ten years, and 80% at fifteen years after diagnosis. However, debilitating effects associated with the toxicities of cancer treatment were found to drastically reduce the quality of life of cancer survivors^1^. Many chemotherapeutic agents with a wide range of mechanisms of action were found to induce numerous side effects due to a lack of cellular specificity. Of these, chemotherapy-induced cognitive impairment (CICI) experienced by 17% to 75% of breast cancer survivors, from 6 months to 20 years after chemotherapy, is one of the direct consequences of cancer chemotherapy^2^. CICI/Chemobrain/Chemofog is generally referred to as short/long-term memory problems manifested by difficulties concerning learning, attention, executive function, and processing speed^3^. Even though different mechanisms have been proposed to explain the underlying cause of chemobrain, comprehensive detailing of molecular pathways is poorly performed, and so it is often unrecognized and untreated. This may be possible because of different mechanisms attributed by chemotherapeutic agents within the chemotherapy regimen^4,5^.

Neuroinflammation, impaired neurogenesis, and oxidative stress are equally considered contributing factors for CICI^6^. In different preclinical studies, neuroinflammation is often studied as a possible explanatory mechanism causing cognitive deficits due to chemotherapy. The inability of a majority of the chemotherapeutic agents to cross blood–brain barrier (BBB) and their role in inducing pathological and biochemical changes in neurons establishes the communication between peripheral and central cytokines. There is a direct correlation between cognitive impairment and cytokine levels^7,8^. Cyclophosphamide (CPP), Methotrexate (MTX), and 5-Fluorouracil (5-FU) regimen (CMF) is commonly used in early-stage breast cancer disease. CMF therapy is more beneficial in triple-negative breast cancer (TNBC) patients and found to be more effective than anthracycline or taxane-based regimens^9^. TNBC is mainly characterized by poor prognosis because of the non-availability of standard treatment where chemotherapy forms the core systemic medical treatment as the patients do not benefit from hormonal or trastuzumab based therapies because of the absence of estrogen (ER) receptor, progesterone (PR) and human epidermal receptor 2 (Her2)^10^. Neuropsychological tests performed in breast cancer survivors exposed to CMF therapy showed lower cognitive performance than random population control and the effects were found to be long-lasting^11^. CPP & 5-FU crosses BBB^12,13^, but the possibility of MTX to reach brain is low. However, all these agents have individually proved to cause neuroinflammation and cognitive impairment in different preclinical studies. Based on our preliminary laboratory findings, a combination of CPP + MTX + 5-FU in healthy Swiss Albino mice showed impairment in learning and memory, which formed the basis of this study to analyze the role of inflammatory cytokines in inducing cognitive impairment in tumor-bearing animals^14^.

No therapeutic interventions are approved for treating chemobrain. But ethnopharmacological studies have documented numerous active constituents isolated from plant sources which postpone neurodegeneration and improve cognitive function^15^. In the present study, Mulmina Mango (marketed ayurvedic formulation) which consists of Mangifera indica fruit pulp, Centella asiatica, Curcuma longa rhizome, and nutrients and vitamins was analyzed against CMF induced cognitive impairment in a mouse model of mammary carcinoma without compromising the anticancer potential of CMF therapy. Hence, the study is designed to understand the neuroprotective role of Mulmina and to decipher its behavioral, biochemical and neuroinflammatory alterations in tumor-bearing animals.

## Materials and methods

### Cell culture and maintenance

The TNBC cell line 4T1 (murine mammary carcinoma cell line derived from BALB/cf3H mice) was obtained from the Elabscience (EP-CL-007), United States and cultured in sterile conditions in Dulbecco's Modified Eagle Medium (DMEM, gibco Life Technologies, Thermo Scientific, South America (12,800–017)) supplemented with 10% foetal bovine serum (gibco Life Technologies, Thermo Scientific, South America (10,270)) and 0.1 percent Anti-anti (antibiotic–antimycotic (15,240–062)) and maintained in a 37 °C, humidified incubator with 5% CO_2_ until the flasks reached 80% confluency. The cell concentration in suspension was set to 1 × 10^6^ cells/ml and was used to induce breast cancer by injecting 0.2 ml into the breast pads^16,17^.

### Experimental animals

Forty-eight female BALB/C mice (8–10-week-old, 25-30g) were collected from Central Animal Research Facility, Manipal, Karnataka (India). Mice were retained in sterile polypropylene cages under controlled conditions (temperature 23 ± 2ºC and humidity 50 ± 5%) with a 12 h light/dark period and kept with dried pellets and water ad libitum. The experimental protocol entitled 'Evaluation of mango-based beverage for its protective effect against chemotherapy-induced cognitive disorders in 4T1-cells induced breast cancer in BALB/C mice' (IAEC approval no: IAEC/KMC/104/2019) was approved by the Institutional Animal Ethics Committee (IAEC), Kasturba Medical College, MAHE, Manipal, Karnataka, India. The studies were carried out in conformity with the guidelines for the use and care of laboratory animals provided by CPCSEA (Committee for the purpose of control and supervision of experiments on animals), Government of India, and also confirms that the authors complied with the ARRIVE guidelines.

### Drugs and chemicals

Cyclophosphamide (Endoxan-N), Methotrexate (Biotrexate) and 5-Fluorouracil (Fluracil), Donepezil (Aricept) were obtained from Kasturba Medical College Pharmacy, Manipal. If not stated, analytical grade reagents were employed for the study.

### Induction of mammary cancer using 4T1 cells

**Figure 1:**
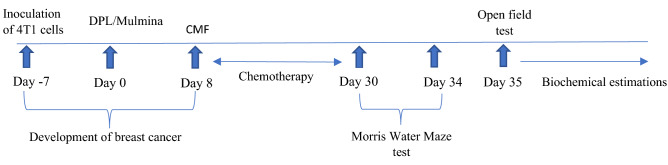
Study design.

Breast cancer induction was done by subcutaneously inoculating 4T1 cells into the mammary fat pad of 8–10-week-old animals. After two weeks, the development of breast cancer was established. The mice were palpated twice a week for the existence of tumors and then randomly assigned into respective groups (n = 8). Intraperitoneal administration of CPP + MTX + 5-FU was carried out once a week for three weeks (21 days). Daily oral treatment of Mulmina (MN- 40,80 ml/ kg, *p.o*) and Donepezil (DPL-2 mg/kg, *p.o*) began one week before chemotherapy and continued till the end of the chemotherapy cycle (Fig: 1).

The health status of the animals was assessed twice daily (morning and evening). Mulmina doses were divided and given at different time points in a day. Chemotherapy involving Cyclophosphamide (morning) and Methotrexate (evening) was administered on the same day, whereas 5-Fluorouracil was given after a one-day interval.

### Experimental groups

**Figure 2:**
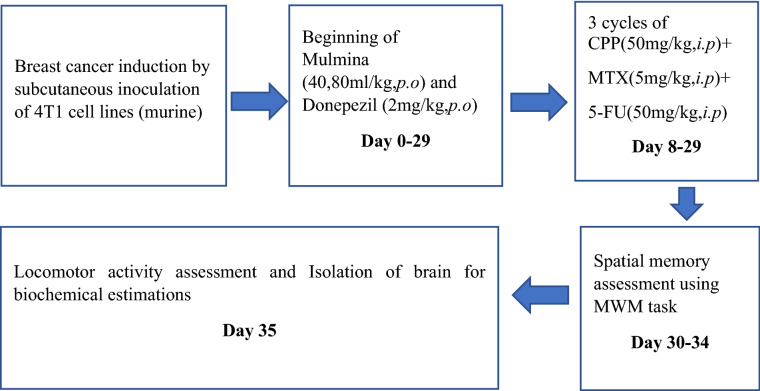
Treatment Schedule.

After cancer induction, the mice were randomly assigned to six different groups (Table 1) and drug treatments were given accordingly (Fig: 2).

**Table 1:** Treatment groups.

Sl. No	Group Name	Treatment	No. of animals
1	Normal control	Carboxy methyl cellulose (10 ml/kg, *p.o*) + Normal saline (1 ml/kg, *i.p*)	8
2	Tumor control	Tumor + Carboxy methyl cellulose (10 ml/kg, *p.o*) + Normal saline (1 ml/kg, *i.p*)	8
3	Tumor + CMF	Tumor + CPP (50 mg/kg, *i.p)*** + **MTX (5 mg/kg, *i.p*) + 5-FU (50 mg/kg, *i.p*)	8
4	Tumor + CMF + DPL	Tumor + CPP (50 mg/kg, *i.p)*** + **MTX (5 mg/kg, *i.p*) + 5-FU (50 mg/kg, *i.p*) + Donepezil (2 mg/kg, *p.o*)	8
5	Tumor + CMF + MN-40	Tumor + CPP (50 mg/kg, *i.p)*** + **MTX (5 mg/kg, *i.p*) + 5-FU (50 mg/kg, *i.p*) + Mulmina (40 ml/kg, *p.o*)	8
6	Tumor + CMF + MN-80	Tumor + CPP (50 mg/kg, *i.p)*** + **MTX (5 mg/kg, *i.p*) + 5-FU (50 mg/kg, *i.p*) + Mulmina (80 ml/kg, *p.o*)	8

Animal doses of Mulmina were selected based on human equivalent dose as per FDA human equivalent dose conversion table. 0.25% Carboxymethylcellulose (CMC) was used as the vehicle for suspending Donepezil and normal saline was used as the vehicle for dissolving Cyclophosphamide.

Behavioral assessments like open-field test (OFT) and Morris Water Maze (MWM) were evaluated after completing three cycles of chemotherapy, i.e., MWM from day 30 to day 34 and OFT on the 35^th^ day. Subsequently, after the behavioral parameters, animals were sacrificed, and brains were isolated for the estimation of antioxidant parameters (malondialdehyde (MDA), catalase and reduced glutathione (GSH)), neuroinflammatory parameters (TNF-α, IL-6, and IL-1β) and neurotrophin, BDNF (brain-derived neurotrophic factor).

### Behavioral assessment

#### Morris water maze test (MWM)

The MWM apparatus is used to evaluate spatial learning and memory. It is organized into four quadrants in a circular tank. A submerged platform was placed 2 cm beneath the water's surface in the target quadrant. Each animal was put through four trials over the course of four days (acquisition trial), with a 30-s break between each session. During acquisition trial, each mouse was placed slowly in the quadrant by holding its face towards the wall of the tank and allowed to swim for 60 s. If the animal was unable to find the platform within 60 s, it was directed and trained to sit on the platform for 30 s. Different parameters such as swimming speed, swimming distance, path efficiency and escape latency (time taken by the animal to reach the target quadrant from the initial quadrant) were recorded. On the fifth day, after a four-day acquisition experiment, a probe/retention trial was conducted. The animal was placed in the quadrant opposite the target quadrant after the removal of hidden platform. Retention time (the amount of time the animal spends in the target quadrant) and escape latency were measured^14^.

### Open-Field test (OFT)

The open-field test was used to examine the animals' locomotor activity and exploratory behaviour. Here the animals were individually placed in the centre of square arena [Clean glass jar (30*30*60 cm) and arena was divided into 9 equal quadrants (10*10 cm)] for the assessment of parameters like number of line crossings, number of centre square entries, latency to first line crossing, time spent in centre square and time spent in the periphery over a 6-min period. The experimental area was cleaned with 70% ethanol after each trial to mask any odour related cues^14^.

### Biochemical assessment

After the behavioral studies, mice were anesthetized with diethyl ether, and blood was withdrawn by retro-orbital puncture to determine different blood parameters. Later, the animals were sacrificed by high-dose anaesthesia followed by cervical dislocation, and the whole brain was collected for different biochemical estimations.

### Tumor progression and tumor volume

Tumor size in the mammary pad was measured to assess the progress of cancer using measuring scale and tumor volume was measured using the formula;

Tumor volume (cm^3^) = Tumor length*(tumor width)^2^.

### Estimation of Haematological parameters

Veterinary blood cell counter (PCE210 VET, ERMA Inc., Tokyo, Japan) was used to estimate various blood parameters such as red blood cells (RBC), white blood cells (WBC), monocytes, lymphocytes, granulocytes, haemoglobin, and platelets.

### Protein estimation

BCA protein assay kit (Bicinchoninic acid) was employed to determine the protein present in tissue samples provided by Cyanagen (QPRO), Italy where bovine serum albumin (BSA) was used as the standard (2 mg/ml).

### Estimation of malondialdehyde (MDA)^18^

The amount of lipid peroxidation was determined using Konings and Drijver's^18^ method. The procedure begins with the preparation of the TBA-TCA-HCl reagent, which is followed by the addition of equal parts reagent and brain homogenate. After that, it was warmed in a boiling water bath for 15 min at 80 °C. The resultant reaction mixture was then centrifuged at 10000 rpm for 10 min at 4ºC. The amount of malondialdehyde formed was measured at absorbance 532 nm using ELISA plate reader.

### Estimation of reduced glutathione (GSH)^19^

The levels of GSH were determined by the procedure given by Moron et. al. 1979 ^19^. Equal amounts of tissue homogenate and TCA reagent solution were centrifuged, and the supernatant was collected. This was followed by incubating supernatant, phosphate buffer, and DTNB for 10 min, after which absorbance was measured at 412 nm using an ELISA plate reader.

### Estimation of catalase^20^

Catalase activity was estimated based on the procedure given by Aebi et.al. 1984. A total of 16.6 µl supernatant was added to the cuvette containing 1 ml of phosphate buffer (pH:7)-H_2_O_2_ solution. The reaction was recorded for 1 min at absorbance 240 nm to measure the decomposition rate of H_2_O_2_ spectrophotometrically. The catalase activity was measured as micromoles of hydrogen peroxide decomposed/minute/milligram of protein.

### Estimation of neuroinflammatory cytokines and neuronal growth factors like BDNF

The levels of TNF-α, IL-1β, IL-6, and BDNF were estimated by utilizing mouse kits obtained from Thermo Fisher Scientific. These immune assays were based on the sandwich principle of ELISA with the help of a microplate reader at 450 nm. Then each sample concentration of TNF-α, IL-1β, IL-6, and BDNF was determined from the standard curve.

### Statistical analysis

The data were analysed statistically using the GraphPad Prism 8.0 software and expressed as mean ± SEM. Except for acquisition trial data, all behavioural and biochemical parameters were assessed using one-way analysis of variance (ANOVA) and Dunnet's post-hoc test. The results from the acquisition trials were analysed using two-way analysis of variance and Bonferroni's post-hoc test. Unpaired student 't' test was also applied in the analysis of oxidative markers, BDNF and inflammatory parameters. At 95 percent confidence interval, *p* < 0.05 was considered statistically significant.

## Results

### Effect of test drugs on Morris water maze activity in tumor-bearing animals

**Figure 3:**
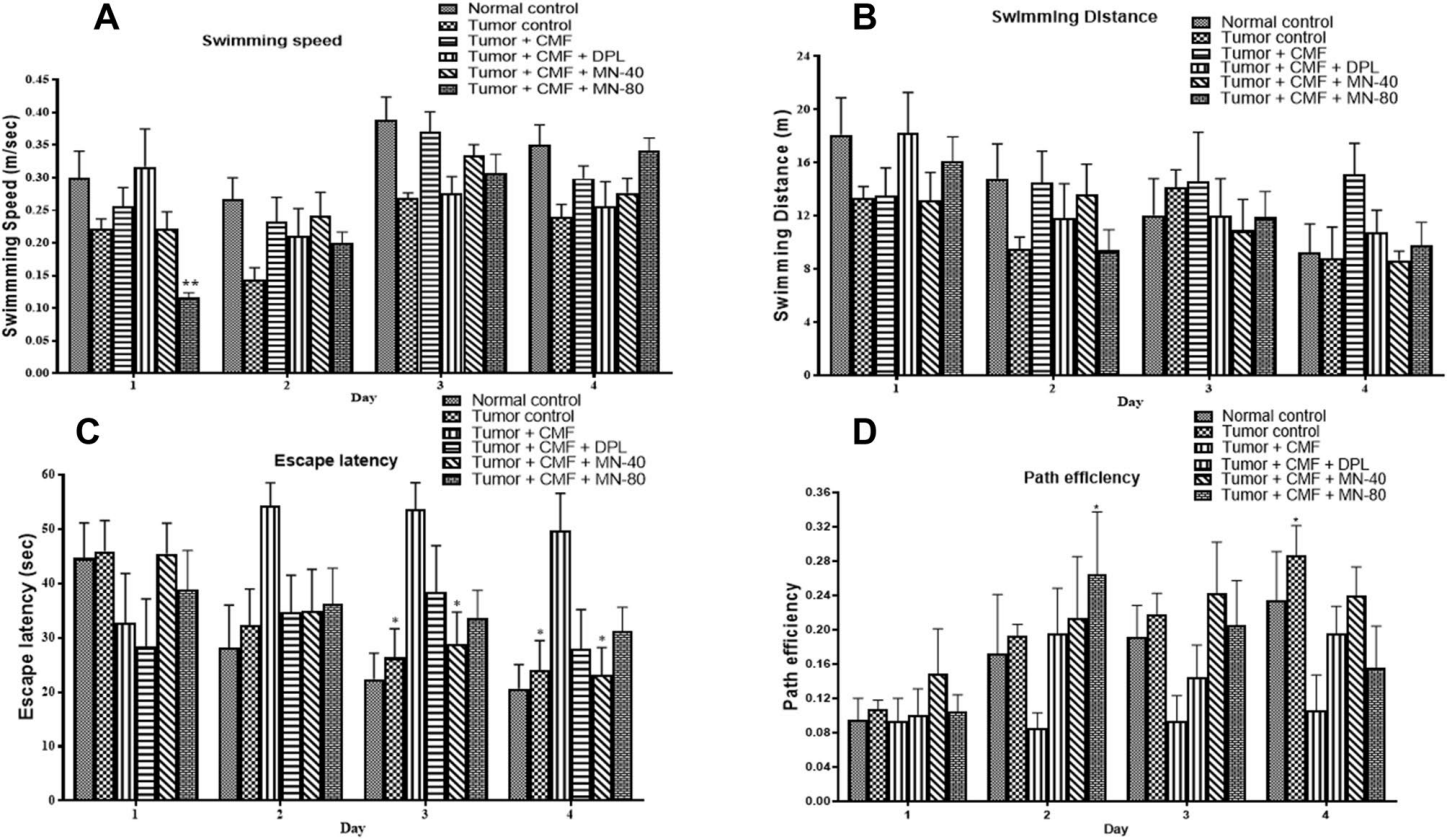
Effect of Mulmina (MN), Cyclophosphamide + Methotrexate + 5-Fluorouracil (CMF), Donepezil (DPL) on (**A**) Swimming speed (**B**) Swimming distance (**C**) Escape latency (**D**) Path efficiency on tumor-bearing animals in MWM acquisition trial. Values are expressed as mean ± SEM (n = 3–4). Statistical analysis was done by using two-way ANOVA followed by Bonferroni's post-hoc test .*p < 0.05,**p < 0.01 compared to tumor + CMF treated group.

**Figure 4:**
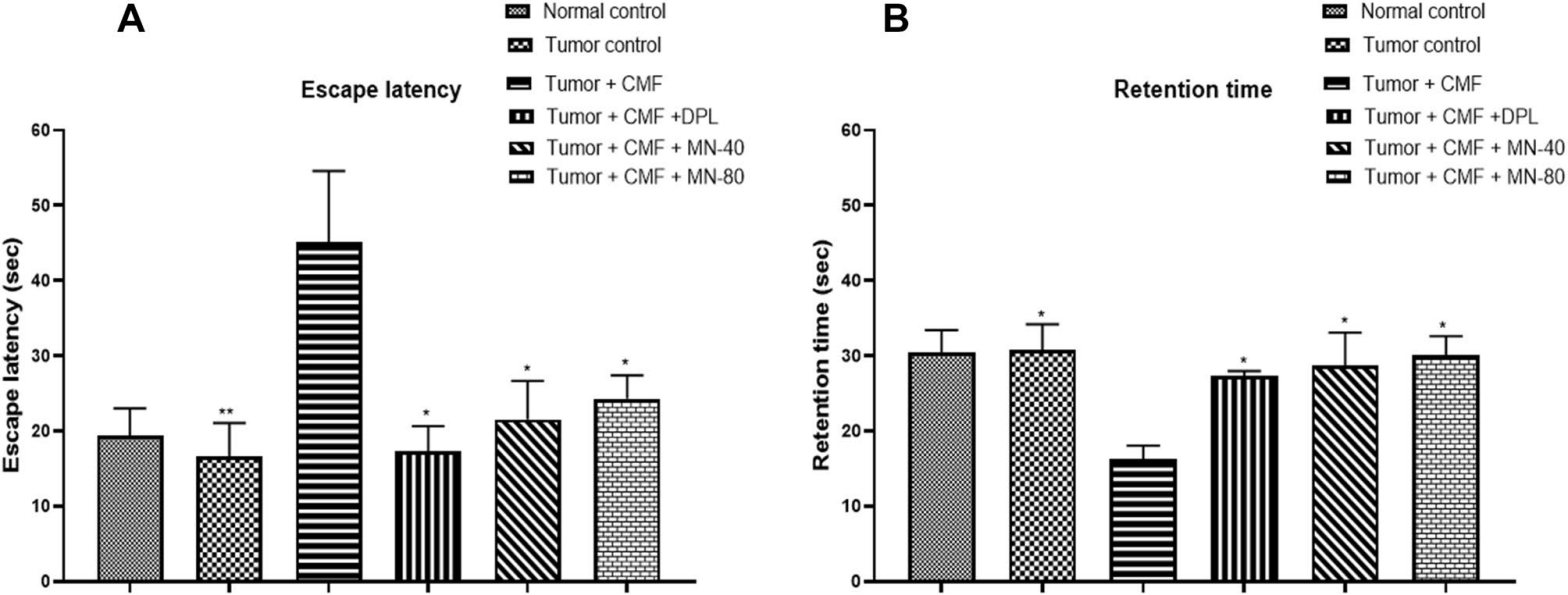
Effect of Mulmina (MN), Cyclophosphamide + Methotrexate + 5-Fluorouracil (CMF), Donepezil (DPL) on (**A**) Escape latency (**B**) Retention time on tumor-bearing animals in MWM retention trial . Values are expressed as mean ± SEM (n = 3–4). Statistical analysis was done by using one-way ANOVA followed by Dunnet's post-hoc test .*p < 0.05,**p < 0.01 compared to tumor + CMF treated group.

During the acquisition trial, CMF treated tumor-bearing animals exhibited a significant impairment in learning, shown by an increase in escape latency and decrease in path efficiency. No significant difference between the groups was observed in swimming distance. But a significant decrease in the swimming speed was found in MN-80 treated group on the first day of acquisition trial when compared to tumor + CMF treated group. There was no significant difference in the learning parameters between normal and tumor control animals. Administration of lower dose of Mulmina (40 ml/Kg) significantly attenuated CMF induced cognitive impairment evidenced by a decrease in escape latency on days 3 and 4 and higher dose of Mulmina (80 ml/Kg) increased path efficiency on day 2 compared to tumor + CMF treated group (Fig: 3). During the retention trial, memory deficits were also improved by both higher and lower doses of Mulmina compared to the tumor + CMF treated group through an increase in the retention time and decrease in escape latency. Additionally, donepezil treatment also enhanced retention time and decreased escape latency when compared to the tumor + CMF treated group (Fig: 4).

### Effect of test drugs on locomotor activity in tumor-bearing animals

**Figure 5:**
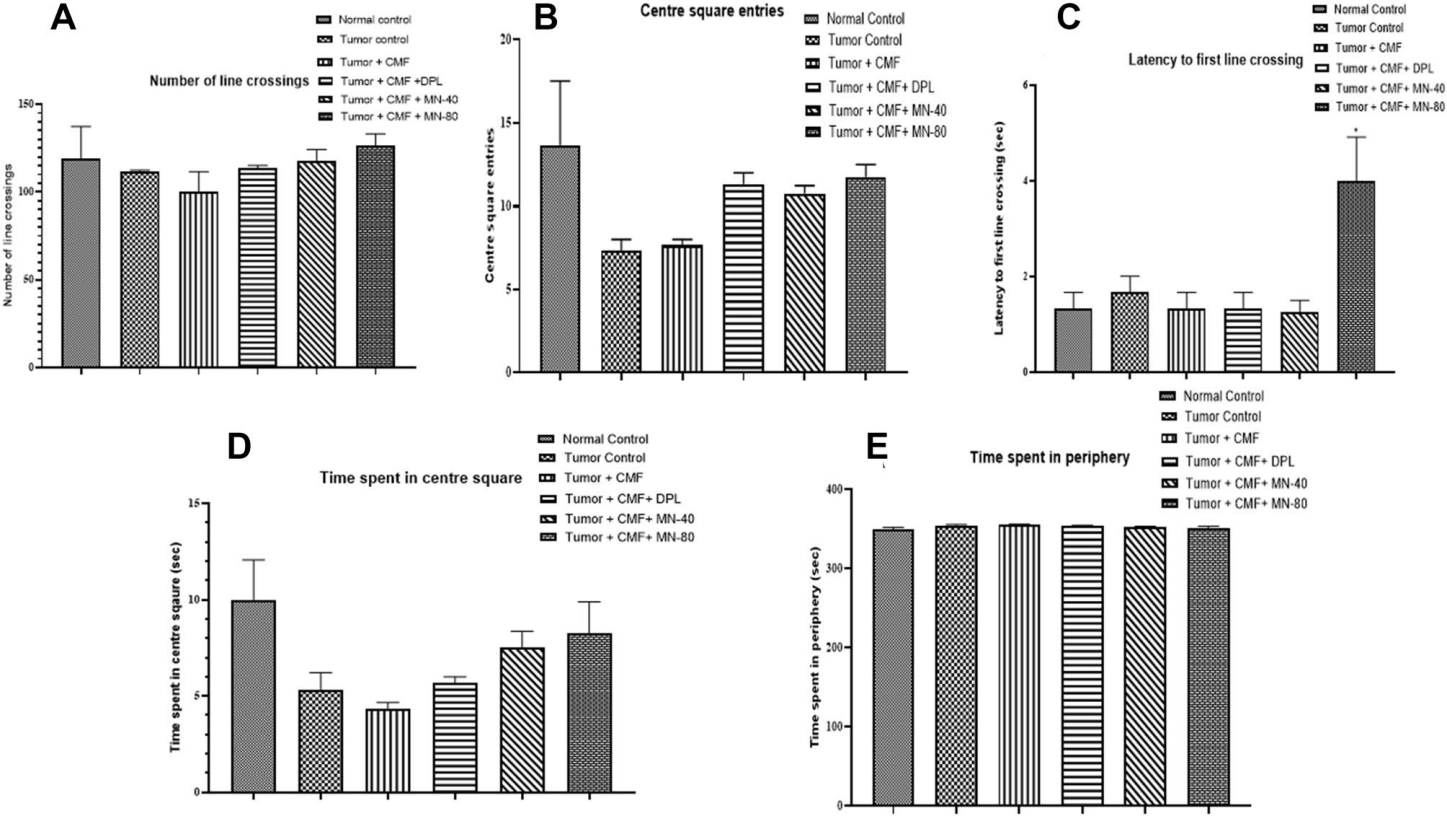
Effect of Mulmina (MN), Cyclophosphamide + Methotrexate + 5-Fluorouracil (CMF), Donepezil (DPL) on (**A**) Number of line crossings (**B**) Centre square entries (**C**) Latency to first line crossing (**D**) Time spent in centre square (**E**) Time spent in periphery on tumor-bearing animals in open-field test. Values are expressed as mean ± SEM (n = 3–4). Statistical analysis was done by using one-way ANOVA followed by Dunnet's post-hoc test .*p < 0.05 compared to tumor + CMF treated group.

Different parameters such as number of line crossings (total number of lines crossed by each animal in the square area which is divided into 9 equal squares), centre square entries (measure of number of times an animal enters into the centre squares), latency to first line crossing (measure of time taken by the animal to cross the first line from the centre square for the first time), time spent in centre square (measure of total time spent by the animal in the centre square) and time spent in periphery (measure of total time spent by the animal in the periphery of the open-field area). Except for an increase in the latency to first line crossing in the MN-80 treated group compared to the tumour + CMF treated group, none of the treatment groups revealed significant differences in any of the measures. (Fig. 5).

### Tumor volume

**Figure 6:**
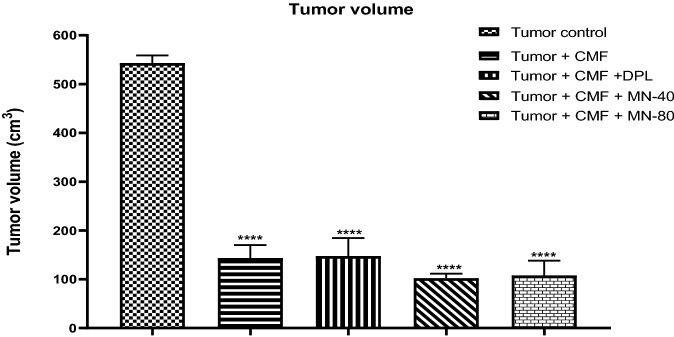
Effect of Mulmina (MN), Cyclophosphamide + Methotrexate + 5-Fluorouracil (CMF), Donepezil (DPL) on tumor volume. Values are expressed as mean ± SEM (n = 3–4). Statistical analysis was done by using one-way ANOVA followed by Dunnet's post-hoc test. ****p < 0.0001 compared to tumor control group.

There was a significant decrease in the tumor volume in all treatment groups (*p* < 0.0001) when compared to the tumor control group (Fig. 6).

### Effect of test drugs on the haematological parameters

**Figure 7:**
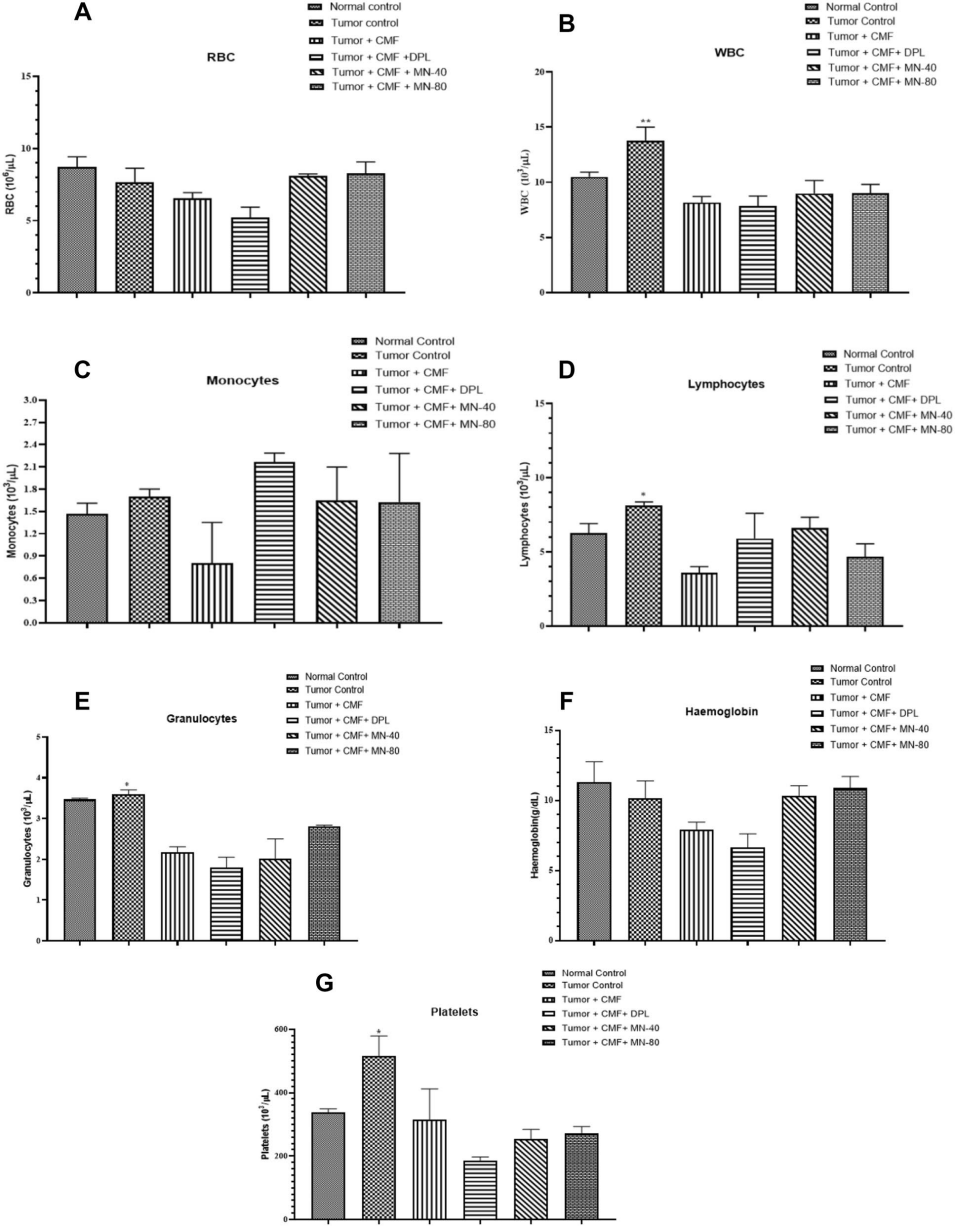
Effect of Mulmina (MN), Cyclophosphamide + Methotrexate + 5-Fluorouracil (CMF), Donepezil (DPL) on **A)** Red blood cell **B)** White blood cell (**C**) Monocytes (**D**) Lymphocytes (**E**) Granulocytes (**F**) Haemoglobin (**G**) Platelets on tumor-bearing animals. Values are expressed as mean ± SEM (n = 3–4). Statistical analysis was done by using one-way ANOVA followed by Dunnet's post-hoc test .*p < 0.05,**p < 0.01 compared to tumor + CMF treated group.

A significant increase in WBC, lymphocytes, granulocytes, and platelets was observed in tumor control group when compared to the tumor + CMF treated group. But changes in RBC, monocytes, and haemoglobin between the groups were found to be statistically insignificant (Fig. 7).

### Effect of test drugs on the level of oxidative stress in brain

#### Lipid peroxidation (LPO), Reduced glutathione (GSH) and Catalase

**Figure 8:**
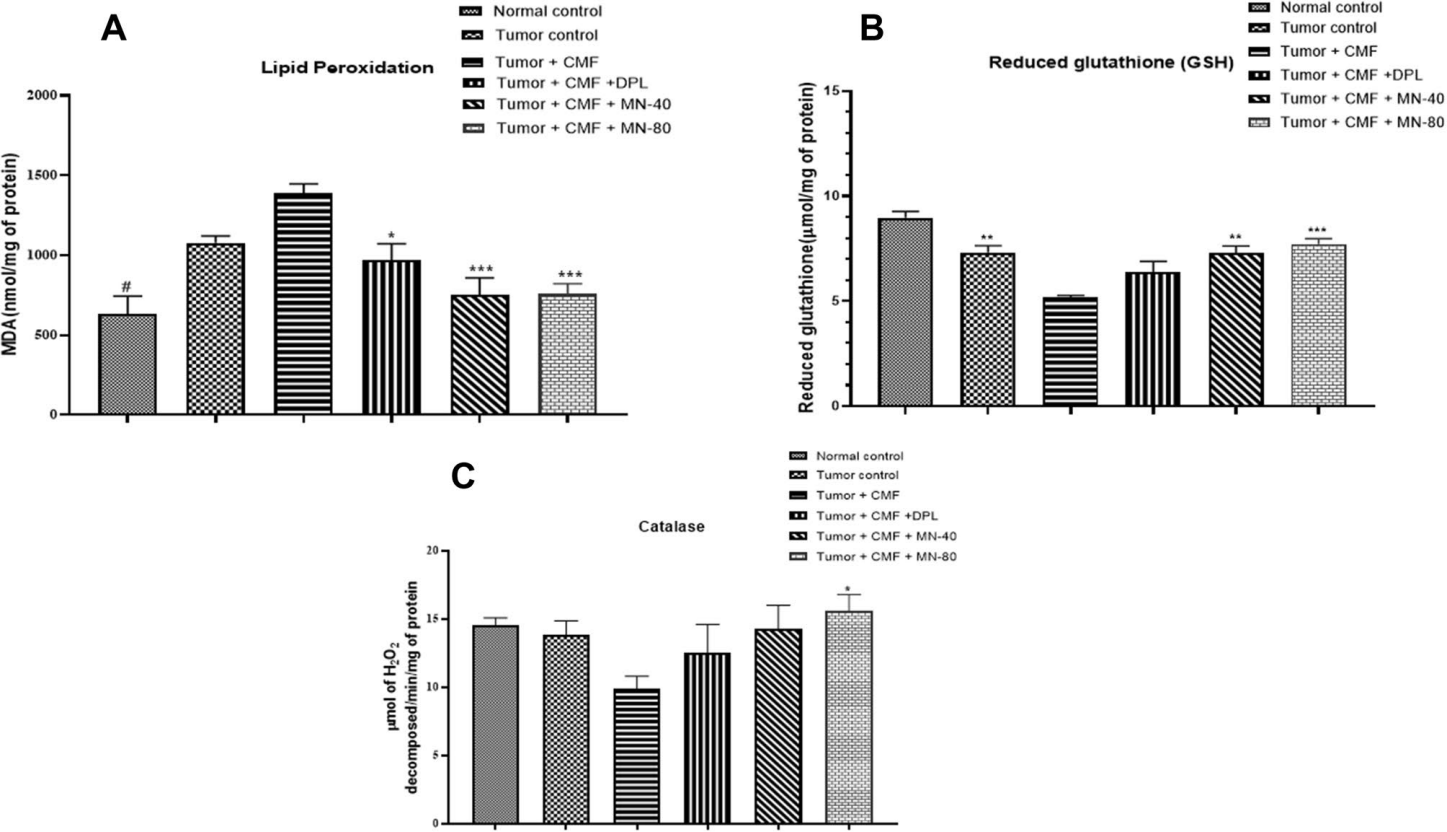
Effect of Mulmina (MN), Cyclophosphamide + Methotrexate + 5-Fluorouracil (CMF), Donepezil (DPL) on (**A**) LPO (**B**) GSH (**C**) Catalase on tumor-bearing animals. Values are expressed as mean ± SEM (n = 3–4). Statistical analysis was done by using one-way anova followed by Dunnet's post-hoc test .*p < 0.05,**p < 0.01, ***p < 0.001 compared to tumor + CMF treated group. #p < 0.05 compared to tumor control group analysed by unpaired student 't' test.

 A significant increase in malondialdehyde levels was observed in the tumor control group (*p* < 0.05) compared to the normal control group. This was further increased in the tumor + CMF treated group due to CMF therapy, where the oxidative stress was found to be more in CMF treated animals than the tumor alone animals. Also, standard DPL (*p* < 0.05) and test treatments MN-40 (*p* < 0.001) and MN-80 (*p* < 0.001) were found to decrease the malondialdehyde (Fig:8A) levels when compared to the tumor + CMF group showing the effectiveness of treatment in reversing the induced oxidative stress by CMF therapy. Similarly, antioxidants like GSH (Fig: 8B) and catalase (Fig: 8C) levels were found to be higher in normal and tumor control animals. But the levels were reduced significantly as a result of CMF therapy in tumor animals. So, the depleted levels of GSH and catalase as a result of CMF therapy in tumor animals (*p* < 0.01) was restored by all the treatment groups but more significantly by the Mulmina treatment, MN-40 (*p* < 0.01) and MN-80 (p < 0.001) (Fig:8).

### Effect of test drugs on neuroinflammatory cytokines like IL-6, IL-1β, and TNF-α and neuronal growth factor like BDNF

#### IL-6 (Interleukin-6), IL-1β (Interleukin-1β), TNF-α (Tumor Necrosis Factor-α) and BDNF (Brain derived neurotrophic factor)

**Figure 9:**
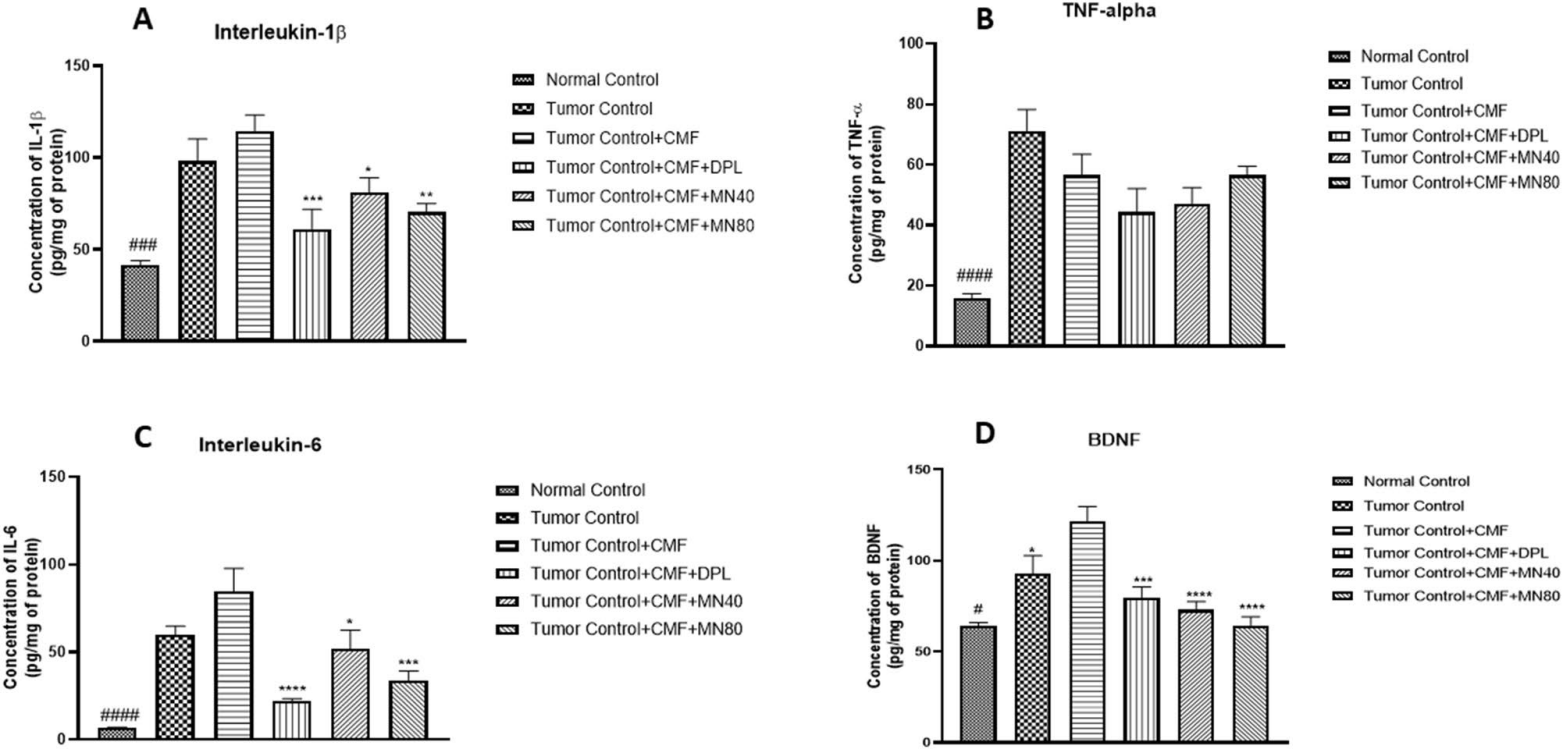
Effect of Mulmina (MN), Cyclophosphamide + Methotrexate + 5-Fluorouracil (CMF), Donepezil (DPL) on **A)** IL-1β **B)** TNF-α **C)** IL-6 **D)** BDNF on tumor-bearing animals. Values are expressed as mean ± SEM (n = 3–4). Statistical analysis was done by using one-way ANOVA followed by Dunnet's post-hoc test .*p < 0.05,**p < 0.01, ***p < 0.001,****p < 0.0001 compared to tumor + CMF treated group.#p<0.05, ###p<0.001, ####p<0.0001 compared to tumor control group analysed by unpaired student 't' test.

Pro-inflammatory cytokines IL-6, IL-1β and TNF-α were found to be higher in tumor-control group compared to normal control animals. We observed a significant decrease in the IL-1β levels in Donepezil (*p* < 0.001), MN-40(p<0.05) and MN-80 (*p* < 0.01) treated groups when compared to the tumor + CMF treated group (Fig: 9A). Also a reduction in the levels of IL-6 was observed in Donepezil (*p* < 0.0001), and both doses of Mulmina, MN-40 (p < 0.05), MN-80 (*p* < 0.001) (Fig:9C).TNF-α levels were also decreased in the treatment groups, but the differences were not statistically significant compared to the tumor + CMF group (Fig:9B). Elevated BDNF (Fig:9D) levels were observed in the tumor control group compared to the normal control group (*p* < 0.05). But the treatment groups, Donepezil (*p* < 0.001), MN-40 (*p* < 0.0001), and MN-80 (*p* < 0.0001) showed a significant decrease in the BDNF levels when compared to the tumor + CMF group showing the protective effect of the treatments, especially that of MN-40 and MN-80 in reversing the enhanced BDNF levels due to chemotherapy (Fig:9).

## Discussion

Chemotherapy-induced cognitive impairment (CICI) is a neuronal injury with inadequate brain repair and abnormal brain remodelling. It is one of the challenges among cancer survivors because it interferes with their day-to-day activities^6^. Chemotherapy regimens are often administered as a cocktail rather than a single agent in the clinical setting, which makes it difficult to dissociate the toxicity conferred by each agent in the regimen^21^. So, the CMF regimen is a well-employed chemotherapeutic approach in breast cancer therapy and found to induce behavioral and biochemical alterations as individual drugs in different preclinical studies^22–25^.

Based on our preliminary findings, we hypothesized that Mulmina Mango, which mainly consists of Mangiferin, could inhibit CMF-induced cognitive impairment in a mouse model of mammary carcinoma. Several studies have documented the anti-inflammatory, antioxidant, and neuroprotective activities of Mangiferin ^26^. The ability of Mangiferin to cross the blood brain barrier makes it an ideal candidate to protect the brain cells from neurotoxicity induced by chemotherapeutic agents. Apart from Mangiferin, asiatic acid from Centella asiatica ^27^ and curcumin ^28^ from Curcuma longa present in Mulmina also contributes to the neuroprotective effects required to reduce the chemotherapeutic toxicity.

The effectiveness of the CMF regimen as a mammary cancer therapy in mice was investigated, and it was found that CMF treatment significantly reduced tumor burden and induced cognitive impairment. We examined tumour volume in all groups except the normal control group and documented that CMF treatment significantly slowed tumour growth when compared to the tumour control group. Pre-treatment of Mulmina (MN-40 & MN-80) and donepezil also showed decreased tumor volume which indicates that Mulmina did not interfered with the anticancer potential of CMF therapy.

Locomotor activity assessment by the open-field test was performed to ensure that results obtained from cognitive assessment tests, especially MWM, are dependent only on the effect of test drugs. We confirmed that all the animals irrespective of groups performed equally in locomotor assessment test with no significant difference between the groups.

Spatial learning and memory were analysed by acquisition and retention trials in the MWM test. We found that on days 3 and 4 of the acquisition trial, escape latency was significantly increased in the CMF treated group, indicating its inability to learn the spatial orientation of the escape platform. Animals treated with a low dose of Mulmina (40 ml/kg, p.o) also showed significant learning behavior than the CMF treated group. Similarly, in the retention trial, the decrease in escape latency and increase in retention time in all the treatment groups(DPL, MN-40, MN-80) signifies the searching behavior and the spatial memory restoration, which was found to be impaired in the CMF group. These changes observed in behavioral parameters shown by CMF therapy are in accordance with the previous studies^14,29,30^.

Regarding haematological parameters, a significant increase observed in WBC count, lymphocyte count, and granulocyte count in the tumor control group compared to tumor + CMF treated group is a usual trend observed in cancer patients treated with chemotherapeutic agents, thereby affecting their immunity^31^. An increased level of platelet count in the tumor control group supports the literature evidence, which states that thrombocytosis can be served as a marker for metastatic breast cancer^32,33^.

Lipid peroxidation, which was found to be higher in both tumor control and CMF treated group indicates the amount of oxidative stress generated in cancer tissues as a result of DNA damage and chemotherapy have a constitutive effect of inducing oxidative stress in tumor cells and multiple cellular targets^34,35^. However, pre-treatment of DPL, MN-40, MN-80 in tumor-bearing animals subjected to chemotherapy protected them from oxidative damage. Glutathione is an antioxidant capable of preventing damage to cellular components caused by reactive oxygen species. Enhanced GSH levels in tumor control group shows increased metabolic activity levels of endogenous antioxidants in tumor cells in the attempt to detoxify from oxidative stress^36^. The pre-treatment with Mulmina restored the disrupted antioxidant status induced by CMF therapy.

Apart from oxidative stress, neuroinflammation also contributes to cognitive deficits. IL-1β, IL-6, and TNF-α are pro-inflammatory cytokines that play an essential role in neuroinflammation^37^. Estimation of common cytokines like IL-6, TNF-α, and IL-1β in brain samples showed a rise in cytokine levels in CMF treated tumor-bearing animals. This finding is in line with the results of a cohort study performed in breast cancer survivors after 20 years of chemotherapy which showed a direct correlation between higher levels of inflammatory markers and lower cognitive functions^38^. The elevated levels of IL-6, IL-1β were reversed significantly by the treatment with Donepezil and both doses of Mulmina. BDNF is a neural growth factor that controls neuronal growth and differentiation^39^. The BDNF levels were found to be higher in CMF alone treated tumor-bearing animals and were significantly reduced in all treatment groups. It could be as a result of long-term activation of microglial cells, which causes the release of BDNF^40^. This finding also agrees with our preliminary study involving normal animals^14^.

As already mentioned, due to the non-availability of drugs for CICI, medications approved for treating other neurological conditions could be employed for providing symptomatic relief to chemobrain patients. Donepezil is an FDA approved drug for treating Alzheimer’s disease. There are different clinical and preclinical studies which proved the suitability of using Donepezil against CICI^41,42^. So in this study, donepezil was taken as a standard therapy for CMF-induced cognitive impairment based on the dose from our preliminary study^14^ and was compared with Mulmina treatment. And we found no significant difference in treatment effects between the donepezil and Mulmina groups.

So, the observed improvement in cognitive function in this study could be attributed to the effect of Mulmina to alleviate the level of neuroinflammatory cytokines and restore antioxidant factors like reduced glutathione and catalase with the reduction in the extent of lipid peroxidation. Our experiment shows that Donepezil and Mulmina prevented CMF induced cognitive impairment in mouse model of mammary carcinoma.

## Conclusion

In this study, the neuroprotective effect of Mulmina against CMF induced cognitive impairment in tumor-bearing mice has been investigated. Mulmina improved cognitive deficits caused by CMF therapy in MWM and other biochemical and neuroinflammatory alterations in the mouse brain without compromising the anticancer potential of CMF treatment. It is concluded that the restoration of cognitive functions by Mulmina may be due to its anti-inflammatory and antioxidant mechanisms. Our study has provided new perceptions of the CMF mechanism that could reveal the effect of Mulmina on cognitive functions. However, elucidation of changes in hepatic enzymes, morphological analysis of brain tissues concerning astrocytosis, microglial activation, and neuronal death due to chemotherapy, and involvement of Mulmina based on these mechanistic perspectives may possibly provide fruitful results in further research.

## Supplementary Information

Supplementary Information.

## Data Availability

The datasets generated during and/or analysed during the current study are available from the corresponding author on reasonable request.

## Abbreviations

**CICI**: Chemotherapy-induced cognitive impairment
**BBB**: Blood–brain barrier
**CPP**: Cyclophosphamide
**MTX**: Methotrexate
**5-FU**: 5-Fluorouracil
**DPL**: Donepezil
**TNBC**: Triple negative breast cancer
**CMF**: Cyclophosphamide + Methotrexate + 5-Fluorouracil
**OFT**: Open-field test
**MWM**: Morris water maze
**MN**: Mulmina
**MDA**: Malondialdehyde
**LPO**: Lipid peroxidation
**GSH**: Reduced glutathione
**TNF-α**: Tumor necrosis factor- α
**IL-6**: Interleukin-6
**IL-1β**: Interleukin-1β
**BDNF**: Brain derived neurotrophic factor
