# Supplementary material for: Neuroprotective effect of Mulmina Mango against chemotherapy-induced cognitive decline in mouse model of mammary carcinoma
Source: Sci Rep. 2022 Feb 23;12:3072. doi: 10.1038/s41598-022-06862-9 (PMC8866531; doi:10.1038/s41598-022-06862-9)
Supplement: Supplementary file 1 — Supplementary Information. [file 41598_2022_6862_MOESM1_ESM.docx]

**Supplementary data**

1. **COMPOSITION OF MULMINA MANGO DRINK**

| **S.N.** | **INGREDIENTS** | **CLAIM/ 200 ML** |
| --- | --- | --- |
| **ACTIVE INGREDIENTS** | | |
| **1** | Blend of Mango pulp | 32 g |
| **2** | Centella asiatica Extract | 40 mg |
| **3** | Turmeric Powder | 100 mg |
| **VITAMINS** | | |
| **4** | Beta-carotene | 400 IU (240 mcg) |
| **5** | Thiamine hydrochloride (Vitamin B1) | 0.2 mg |
| **6** | Riboflavin 5 Phosphate Sodium (Vitamin B2) | 0.15 mg |
| **7** | Nicotinamide (Vitamin B3) | 1.0 mg |
| **8** | Calcium Pantothenate (Vitamin B5) | 0.5 mg |
| **9** | Pyridoxine hydrochloride (Vitamin B6) | 0.15 mg |
| **10** | Folic acid (Vitamin B9) | 80 mcg |
| **11** | Ascorbic acid (Vitamin C) | 40 mg |
| **12** | Tocopheryl acetate (Vitamin E) | 1.0 mg |
| **MINERALS** | | |
| **13** | Calcium lactate Penta hydrate | 25 mg |
| **14** | Ferrous gluconate Anhydrous | 0.5 mg |
| **15** | Magnesium sulphate Heptahydrate | 10 mg |
| **16** | Manganese sulphate | 50 mcg |
| **17** | Zinc Sulphate Heptahydrate | 0.5 mg |
| **18** | Sodium Selenate | 1.5 mcg |
| **ADDITIVES** | | |
| **19** | Sugar | q.s. |
| **20** | Dextrose monohydrate | q.s. |
| **21** | Citric acid monohydrate | q.s. |
| **22** | Pectin | q.s. |
| **23** | Colours  Sunset yellow & Tartrazine | q.s. |
| **24** | Mango flavor | q.s. |
| **25** | Purified Water | q.s. to 200 ml |

Table 1: Composition of Mulmina

**Analytical standardisation data of important constituents of Mulmina Mango using HPLC**

Mulmina® Mango is a novel functional food (nutritional health drinks) which contains medicinal herbs such as Curcuma longa (Turmeric), Centella asiatica (Gotukola), and Mangifera indica (Mango). Mango pulp was blended with other main constituents like Turmeric and Centella asiatica.

**Method validation for asiaticoside content in Mulmina**

Linearity test

Sample: Mulmina Mango (Centella Asiatica)

| **Concentration(%)**  **Asiaticosides** | **Area** |
| --- | --- |
| 80 | 15370 |
| 90 | 17980 |
| 100 | 20785 |
| 110 | 22850 |
| 120 | 24599 |


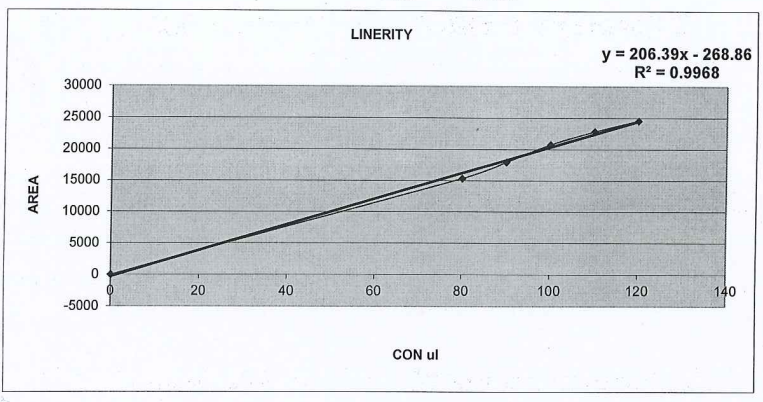


**Asiaticoside standard (80%)**

**
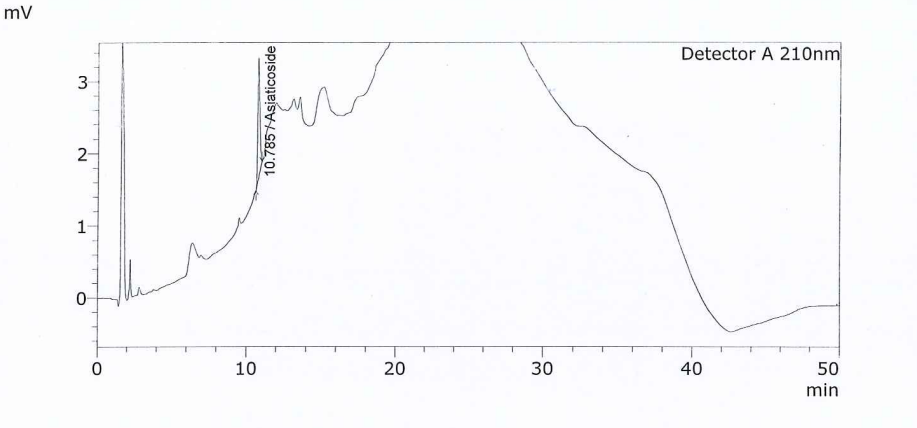
**

| **Peak** | **Name** | **Ret.time** | **Area** | **Height** | **Area** |
| --- | --- | --- | --- | --- | --- |
| 1 | Asiaticoside | 10.785 | 15370 | 1690 | 100% |
| Total |  |  | 15370 | 1690 | 100% |

**Asiaticoside standard (90%)**


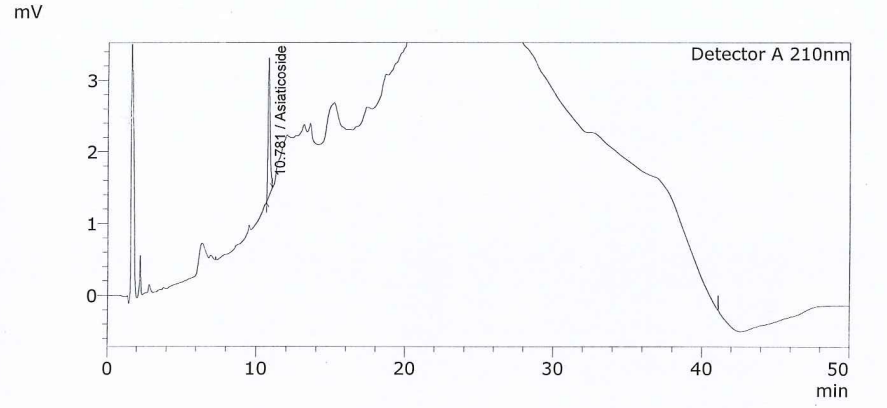


| **Peak** | **Name** | **Ret.time** | **Area** | **Height** | **Area** |
| --- | --- | --- | --- | --- | --- |
| 1 | Asiaticoside | 10.781 | 17980 | 1953 | 100% |
| Total |  |  | 17980 | 1953 | 100% |

**Asiaticoside standard (100%)**


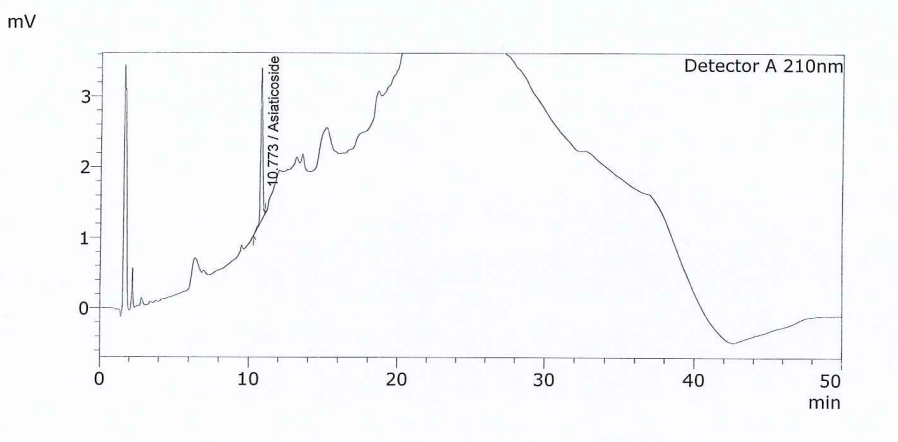


| **Peak** | **Name** | **Ret.time** | **Area** | **Height** | **Area** |
| --- | --- | --- | --- | --- | --- |
| 1 | Asiaticoside | 10.773 | 20785 | 2176 | 100% |
| Total |  |  | 20785 | 2176 | 100% |

**Asiaticoside standard (110%)**


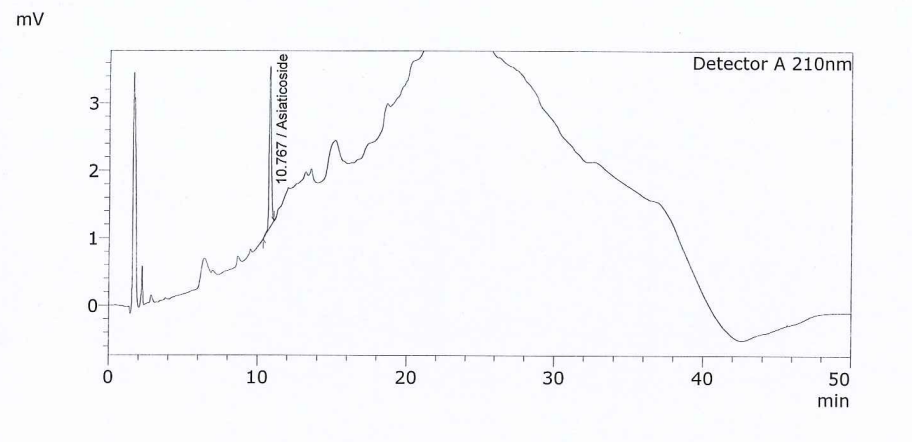


| **Peak** | **Name** | **Ret.time** | **Area** | **Height** | **Area** |
| --- | --- | --- | --- | --- | --- |
| 1 | Asiaticoside | 10.767 | 22850 | 2409 | 100% |
| Total |  |  | 22850 | 2409 | 100% |

**Asiaticoside standard (120%)**


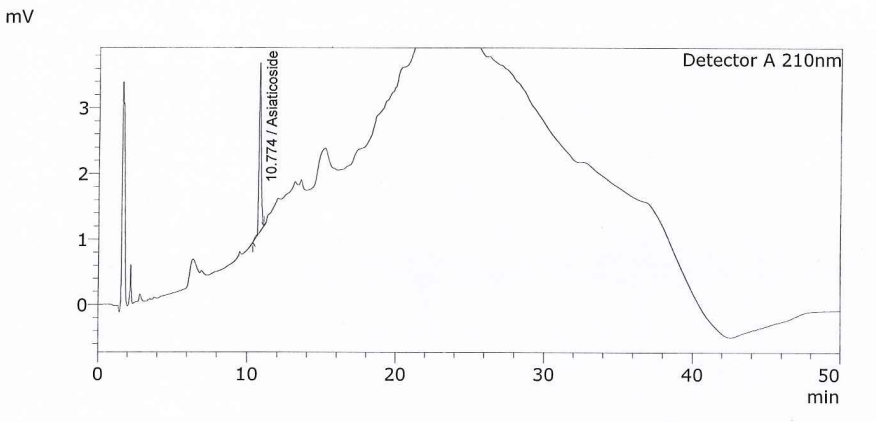


| **Peak** | **Name** | **Ret.time** | **Area** | **Height** | **Area** |
| --- | --- | --- | --- | --- | --- |
| 1 | Asiaticoside | 10.774 | 24599 | 2589 | 100% |
| Total |  |  | 24599 | 2589 | 100% |

**System precision**

| **Injections** | **Area** | **RT** |
| --- | --- | --- |
| 1 | 20805 | 10.771 |
| 2 | 21040 | 10.779 |
| 3 | 20680 | 10.763 |
| 4 | 20551 | 10.767 |
| 5 | 20492 | 10.767 |
| Average | 20714 | 10.769 |
| Standard deviation | 219 | 0.01 |
| % RSD | 1.06 | 0.06 |
|  | NMT | NMT |
| % RSD limit | 2% | 2% |

**Asiaticoside standard 1**


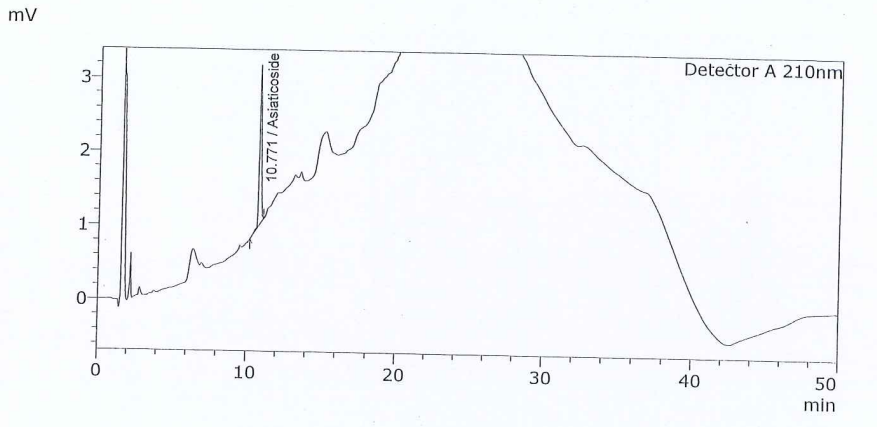


| **Peak** | **Name** | **Ret.time** | **Area** | **Height** | **Area** |
| --- | --- | --- | --- | --- | --- |
| 1 | Asiaticoside | 10.771 | 20805 | 2180 | 100% |
| Total |  |  | 20805 | 2180 | 100% |

**Asiaticoside standard 2**


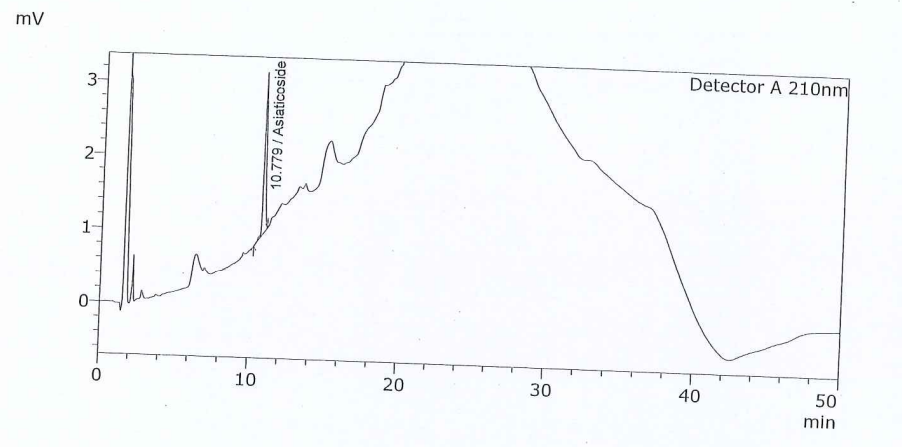


| **Peak** | **Name** | **Ret.time** | **Area** | **Height** | **Area** |
| --- | --- | --- | --- | --- | --- |
| 1 | Asiaticoside | 10.779 | 21040 | 2196 | 100% |
| Total |  |  | 21040 | 2196 | 100% |

**Asiaticoside standard 3**


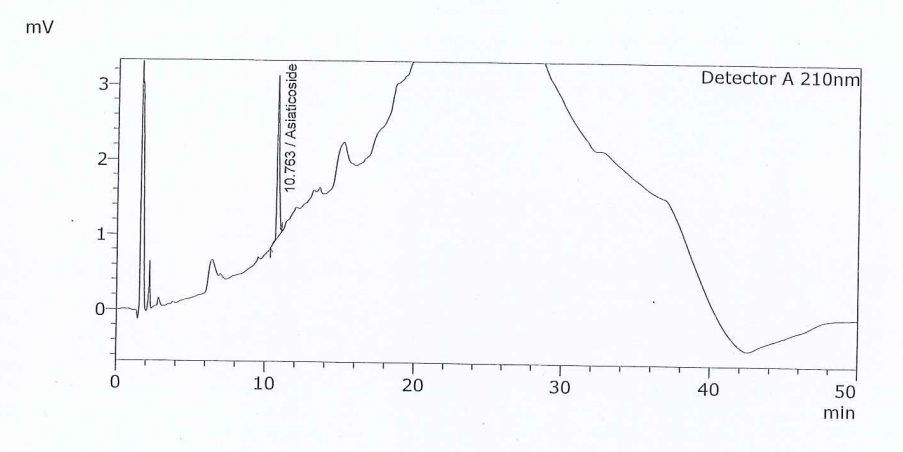


| **Peak** | **Name** | **Ret.time** | **Area** | **Height** | **Area** |
| --- | --- | --- | --- | --- | --- |
| 1 | Asiaticoside | 10.763 | 20680 | 2168 | 100% |
| Total |  |  | 20680 | 2168 | 100% |

**Asiaticoside standard 4**


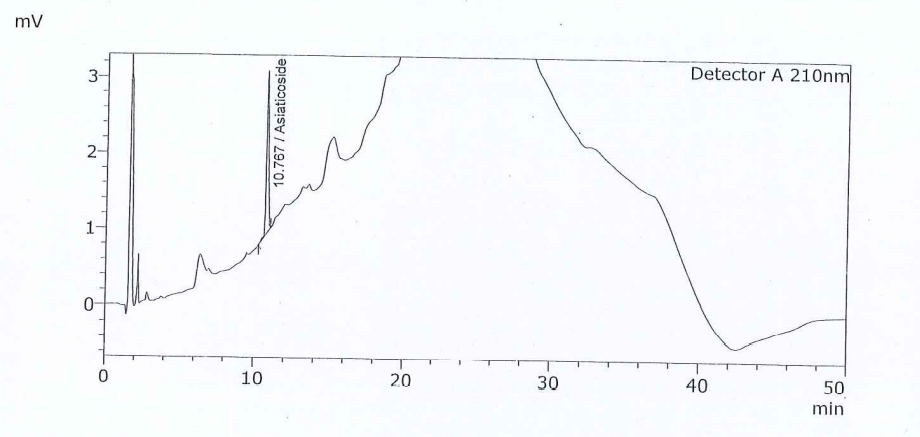


| **Peak** | **Name** | **Ret.time** | **Area** | **Height** | **Area** |
| --- | --- | --- | --- | --- | --- |
| 1 | Asiaticoside | 10.767 | 20551 | 2158 | 100% |
| Total |  |  | 20551 | 2158 | 100% |

**Asiaticoside standard 5**


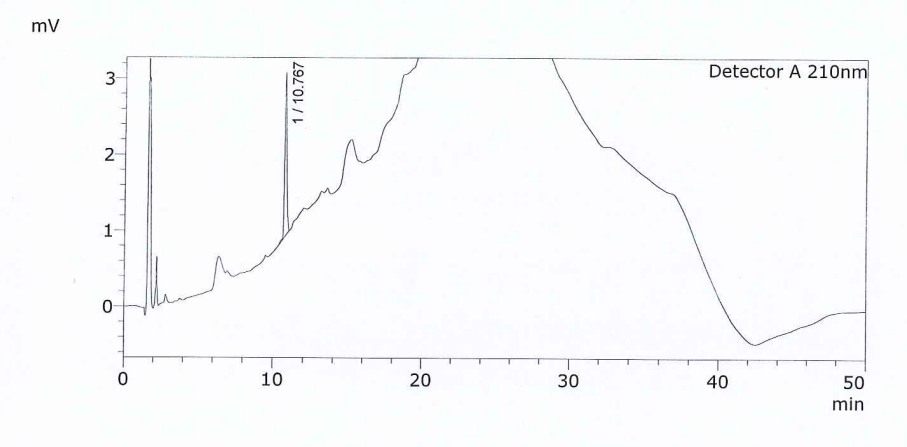


| **Peak** | **Name** | **Ret.time** | **Area** | **Height** | **Area** |
| --- | --- | --- | --- | --- | --- |
| 1 | Asiaticoside | 10.767 | 20492 | 2158 | 100% |
| Total |  |  | 20492 | 2158 | 100% |

| **Time (min)** | **Buffer Conc. Mobile Phase A** | **Acetonitrile Conc. Mobile phase B** |
| --- | --- | --- |
| 0.01 | 85 | 15 |
| 5.0 | 75 | 25 |
| 15 | 55 | 45 |
| 20 | 40 | 60 |
| 25 | 40 | 60 |
| 30 | 50 | 50 |
| 35 | 55 | 45 |
| 40 | 85 | 15 |
| 45 | 85 | 15 |
| 50 | 85 | 15 |

**Results**

| Test Substance | Sample Area | Standard area |
| --- | --- | --- |
| Asiaticosides | 2813 | 20805 |
|  | 2822 | 21040 |
|  |  | 20680 |
|  |  | 20551 |
|  |  | 20492 |
| Average | 2818 | 20714 |

The asiaticosides content in the sample was found to be 0.227mg/200 ml

**Asiaticoside standard 1**


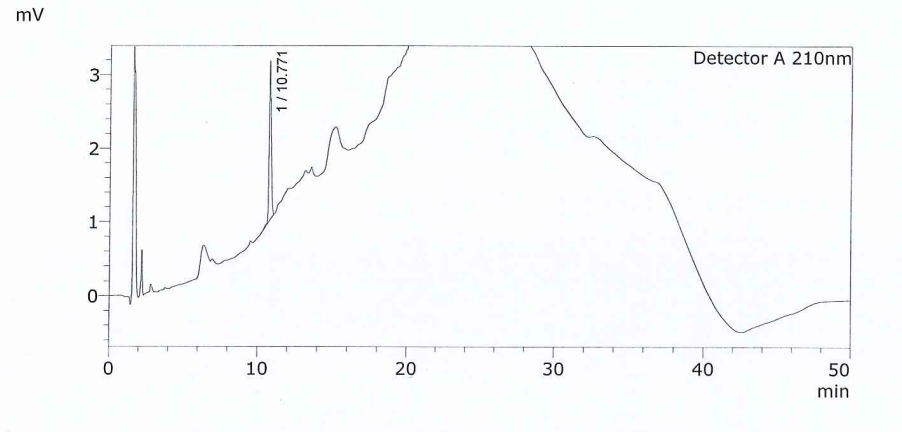


| **Peak** | **Name** | **Ret.time** | **Area** | **Height** | **Area** |
| --- | --- | --- | --- | --- | --- |
| 1 | Asiaticoside | 10.771 | 20805 | 2180 | 100% |
| Total |  |  | 20805 | 2180 | 100% |

**Asiaticoside standard 2**


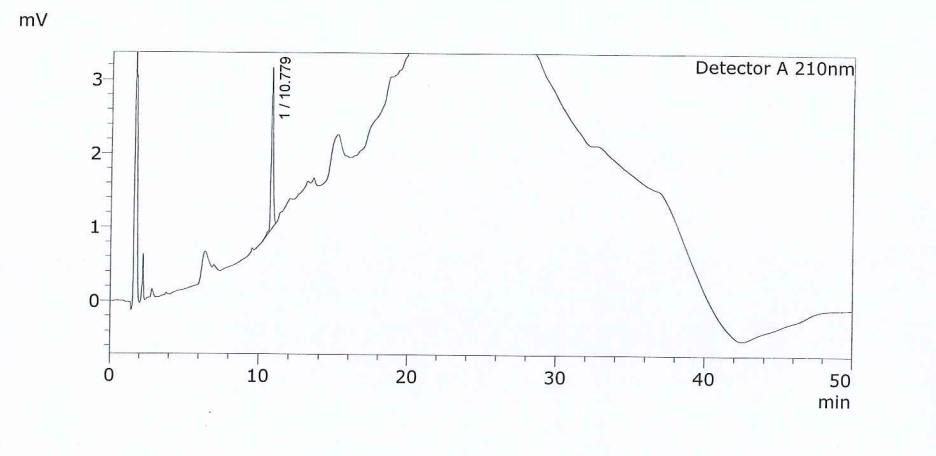


| **Peak** | **Name** | **Ret.time** | **Area** | **Height** | **Area** |
| --- | --- | --- | --- | --- | --- |
| 1 | Asiaticoside | 10.779 | 21040 | 2196 | 100% |
| Total |  |  | 21040 | 2196 | 100% |

**Asiaticoside standard 3**


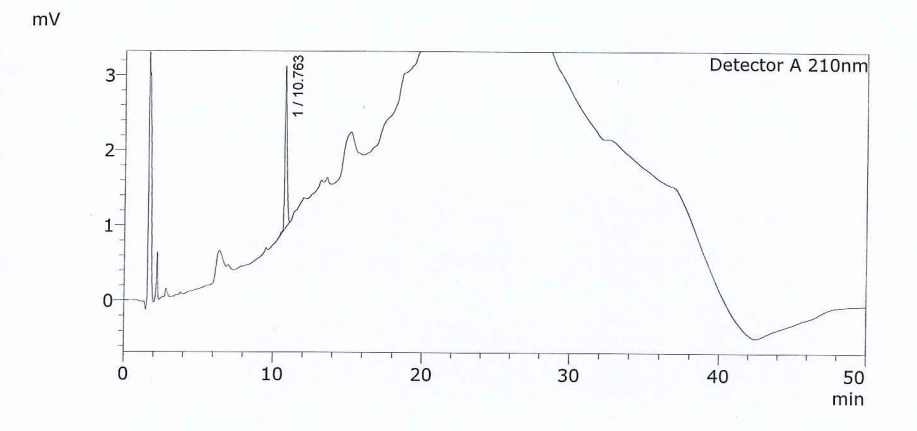


| **Peak** | **Name** | **Ret.time** | **Area** | **Height** | **Area** |
| --- | --- | --- | --- | --- | --- |
| 1 | Asiaticoside | 10.763 | 20680 | 2168 | 100% |
| Total |  |  | 20680 | 2168 | 100% |

**Asiaticoside standard 4**


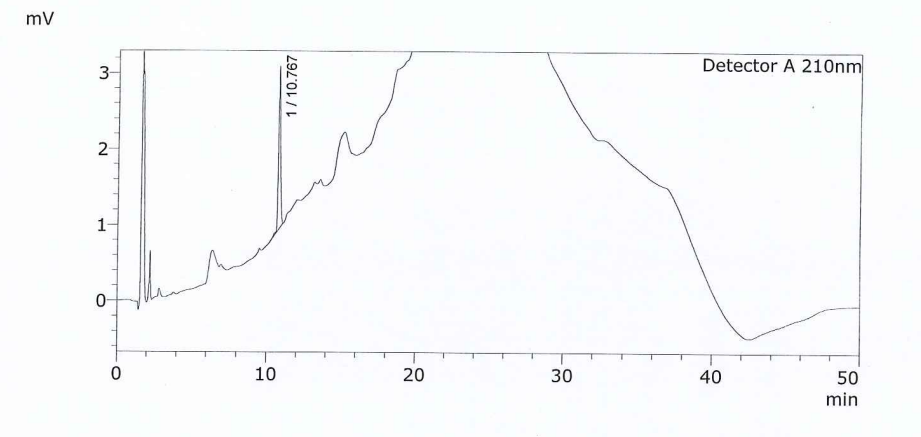


| **Peak** | **Name** | **Ret.time** | **Area** | **Height** | **Area** |
| --- | --- | --- | --- | --- | --- |
| 1 | Asiaticoside | 10.767 | 20551 | 2158 | 100% |
| Total |  |  | 20551 | 2158 | 100% |

**Asiaticoside standard 5**


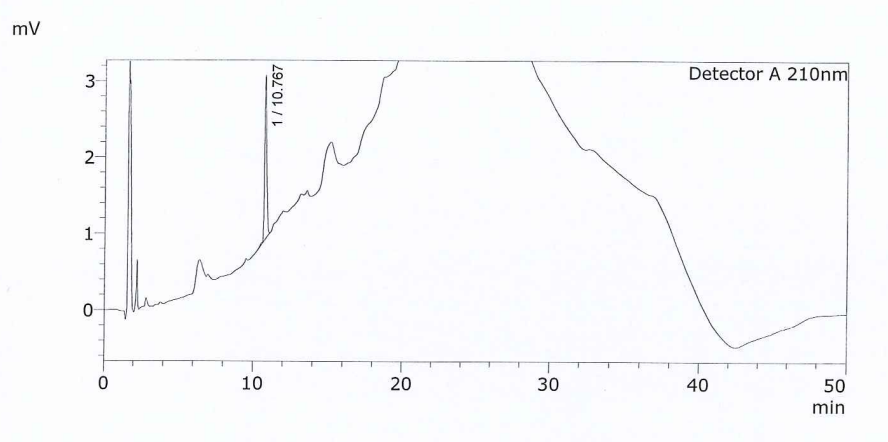


| **Peak** | **Name** | **Ret.time** | **Area** | **Height** | **Area** |
| --- | --- | --- | --- | --- | --- |
| 1 | Asiaticoside | 10.767 | 20492 | 2158 | 100% |
| Total |  |  | 20492 | 2158 | 100% |

**Asiaticoside sample 1**


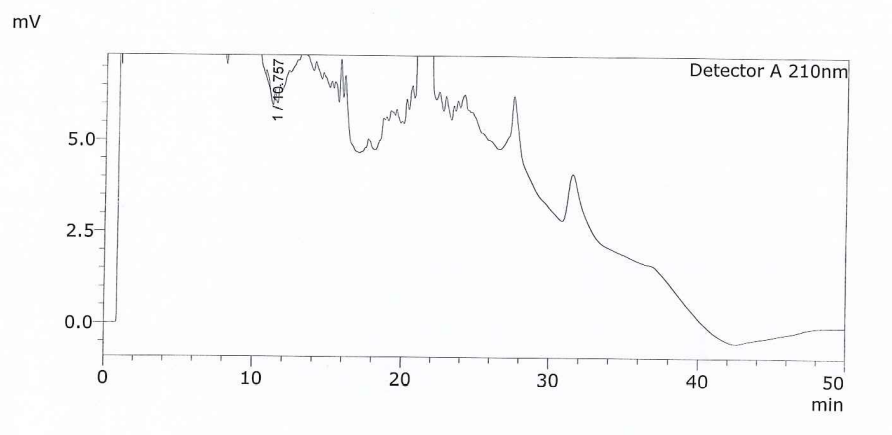


| **Peak** | **Name** | **Ret.time** | **Area** | **Height** | **Area** |
| --- | --- | --- | --- | --- | --- |
| 1 | Asiaticoside | 10.757 | 2813 | 210 | 100% |
| Total |  |  | 2813 | 210 | 100% |

**Asiaticoside sample 2**


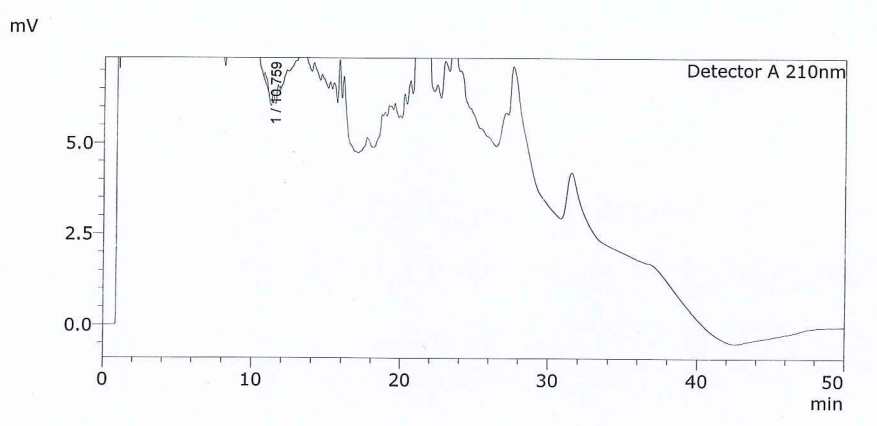


| **Peak** | **Name** | **Ret.time** | **Area** | **Height** | **Area** |
| --- | --- | --- | --- | --- | --- |
| 1 | Asiaticoside | 10.759 | 2822 | 188 | 100% |
| Total |  |  | 2822 | 188 | 100% |

**Results for the analysis of curcumin content in Turmeric powder**

| **Test substance** | **Area** |
| --- | --- |
| Sample | 923115 |
|  | 921653 |
| Average | 922384 |
| Standard | 1054236 |
|  | 1019911 |
|  | 1023319 |
| Average | 1032489 |

The curcumin content in the sample was found to be 3.72%

**Curcumin standard 1**


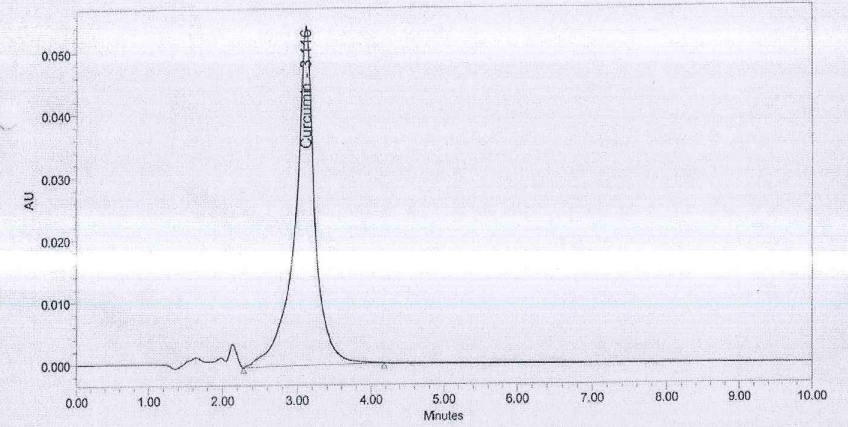


| **Peak Name** | **RT** | **Area** | **% Area** | **Height** |
| --- | --- | --- | --- | --- |
| Curcumin | 3.116 | 1023319 | 100 | 54461 |

**Curcumin standard 2**

**
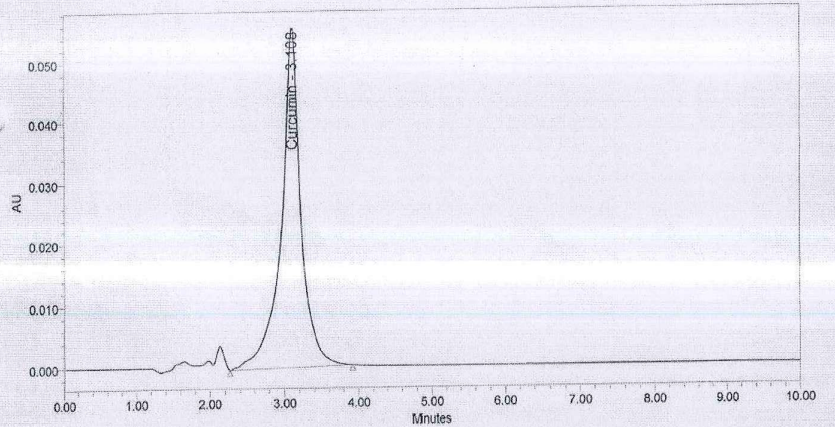
**

| **Peak Name** | **RT** | **Area** | **% Area** | **Height** |
| --- | --- | --- | --- | --- |
| Curcumin | 3.108 | 1019911 | 100 | 55743 |

**Curcumin standard 3**

**
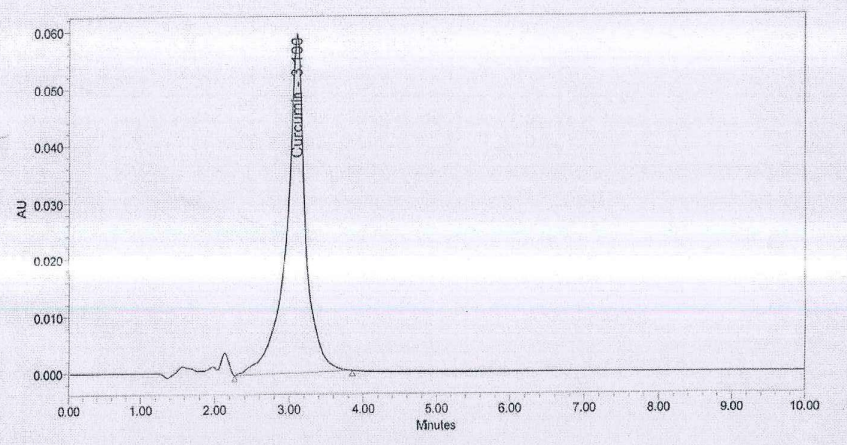
**

| **Peak Name** | **RT** | **Area** | **% Area** | **Height** |
| --- | --- | --- | --- | --- |
| Curcumin | 3.100 | 1054236 | 100 | 59471 |

**Curcumin sample 1**

**
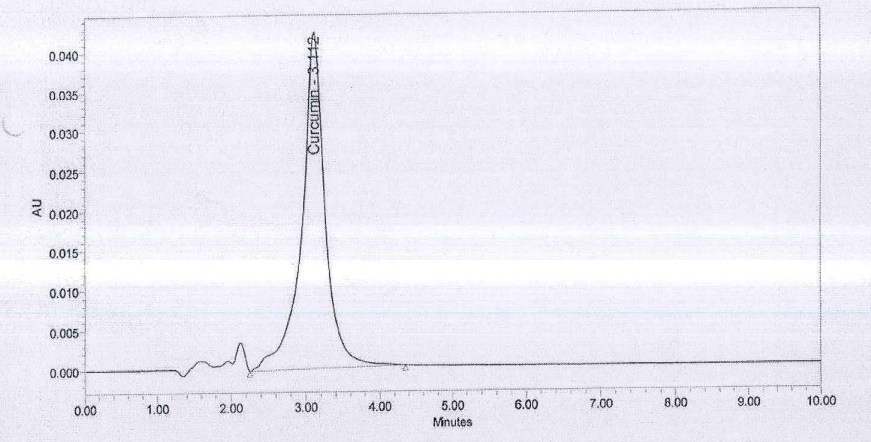
**

| **Peak Name** | **RT** | **Area** | **% Area** | **Height** |
| --- | --- | --- | --- | --- |
| Curcumin | 3.112 | 921653 | 100 | 42458 |

**Curcumin sample 2**

**
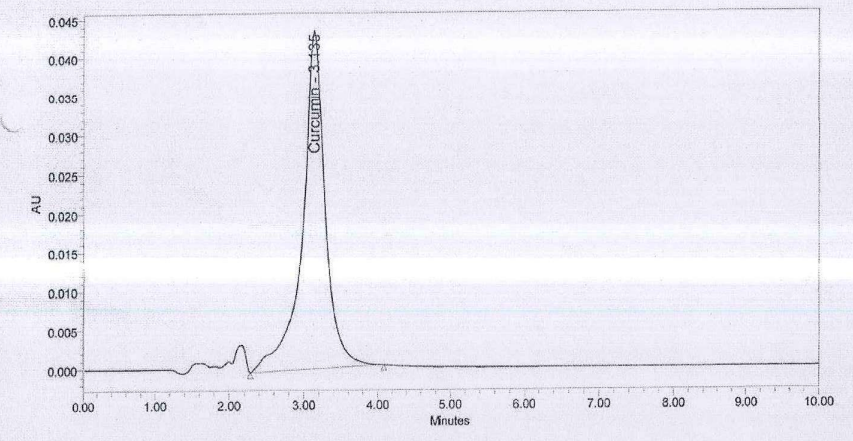
**

| **Peak Name** | **RT** | **Area** | **% Area** | **Height** |
| --- | --- | --- | --- | --- |
| Curcumin | 3.135 | 923115 | 100 | 43484 |

**Method validation for curcumin content**

**Linerarity test**

Sample: Mulmina Mango (Curcumin)

| **Concentration(%)**  **Curcumin** | **Area-1** | **Area-2** | **Average** |
| --- | --- | --- | --- |
| 80 | 1073332 | 1040826 | 1056529 |
| 90 | 1201245 | 1209125 | 1205185 |
| 100 | 1328319 | 1302226 | 1315273 |
| 110 | 1459606 | 1448347 | 1453977 |
| 120 | 1583091 | 1600885 | 1591988 |


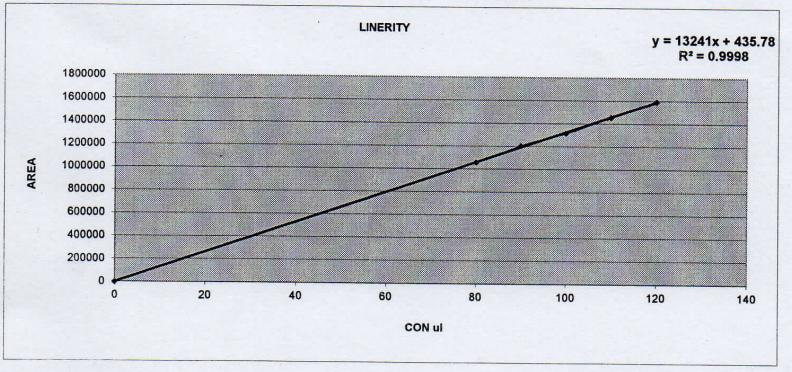


**Curcumin standard 80%**

**
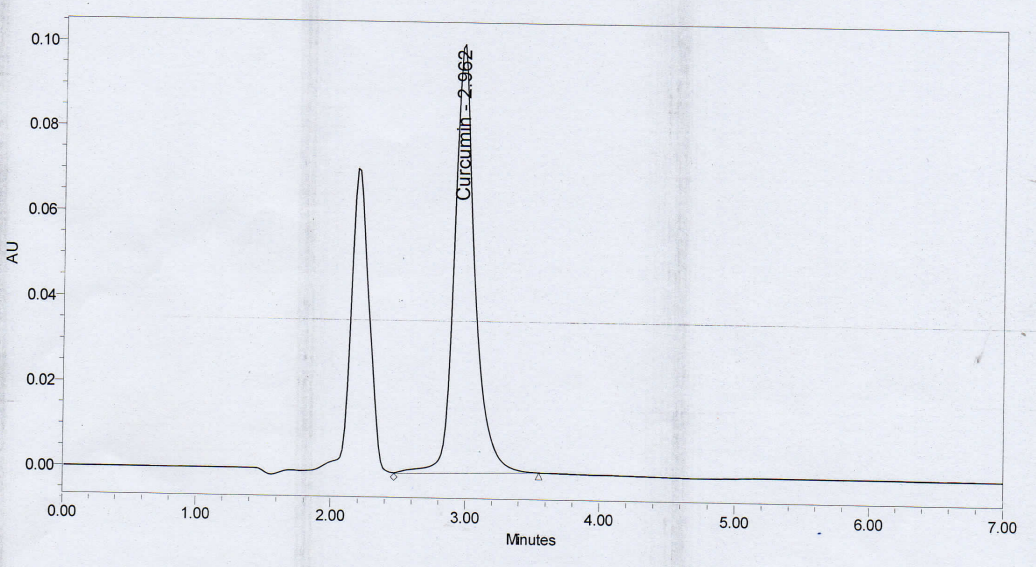
**

| **Peak Name** | **RT** | **Area** | **% Area** | **Height** |
| --- | --- | --- | --- | --- |
| Curcumin | 2.962 | 1072232 | 100 | 100895 |

**Curcumin standard 80%**

**
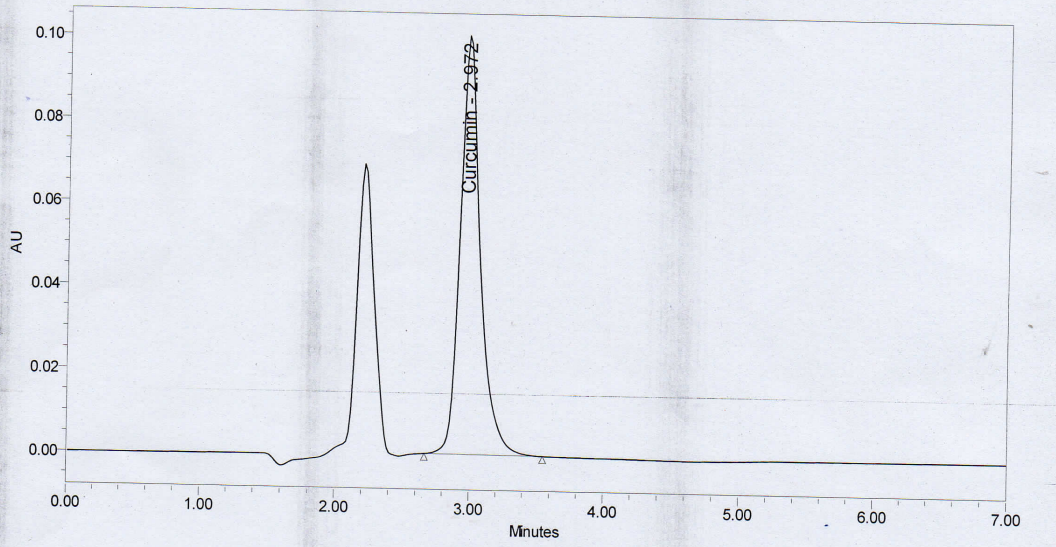
**

| **Peak Name** | **RT** | **Area** | **% Area** | **Height** |
| --- | --- | --- | --- | --- |
| Curcumin | 2.972 | 1040826 | 100 | 100589 |

**Curcumin standard 90%**

**
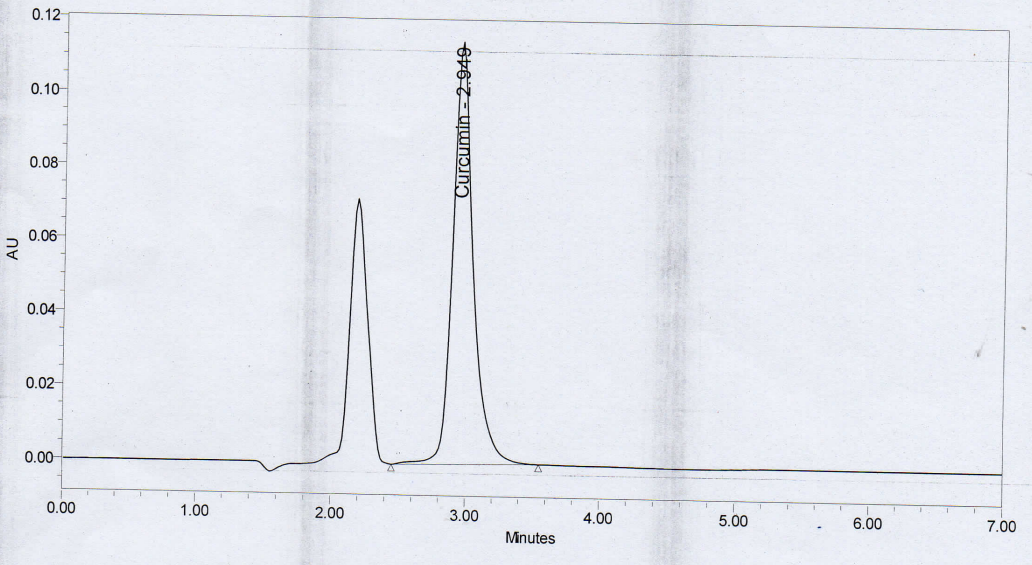
**

| **Peak Name** | **RT** | **Area** | **% Area** | **Height** |
| --- | --- | --- | --- | --- |
| Curcumin | 2.949 | 1201245 | 100 | 114201 |

**Curcumin standard 90%**

**
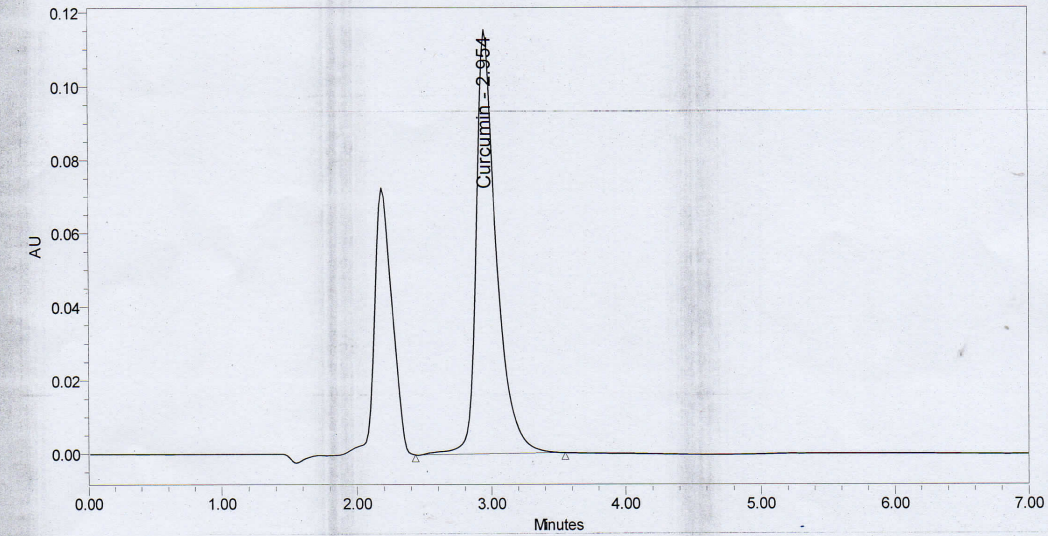
**

| **Peak Name** | **RT** | **Area** | **% Area** | **Height** |
| --- | --- | --- | --- | --- |
| Curcumin | 2.954 | 1209125 | 100 | 115287 |

**Curcumin standard 100%**

**
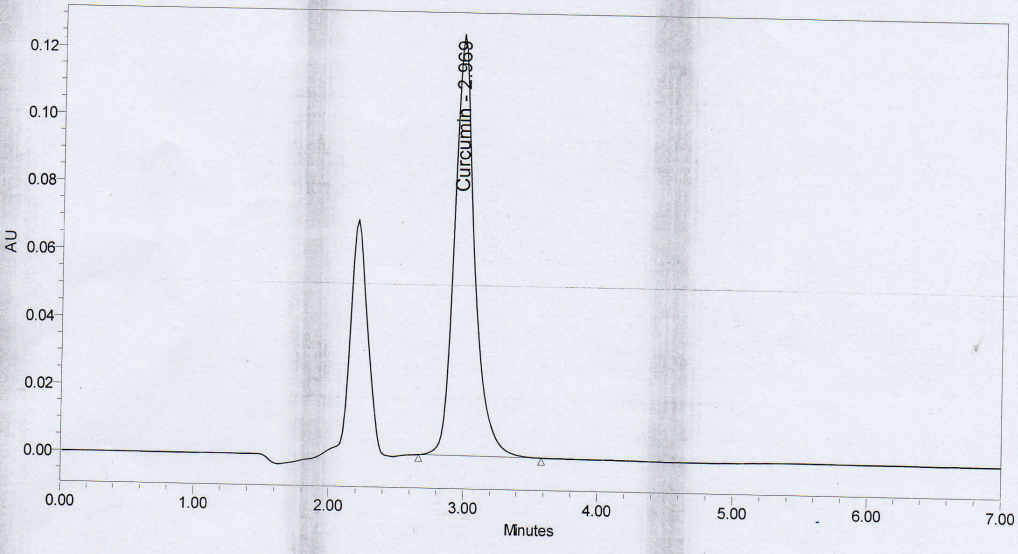
**

| **Peak Name** | **RT** | **Area** | **% Area** | **Height** |
| --- | --- | --- | --- | --- |
| Curcumin | 2.969 | 1302226 | 100 | 124752 |

**Curcumin standard 100%**

**
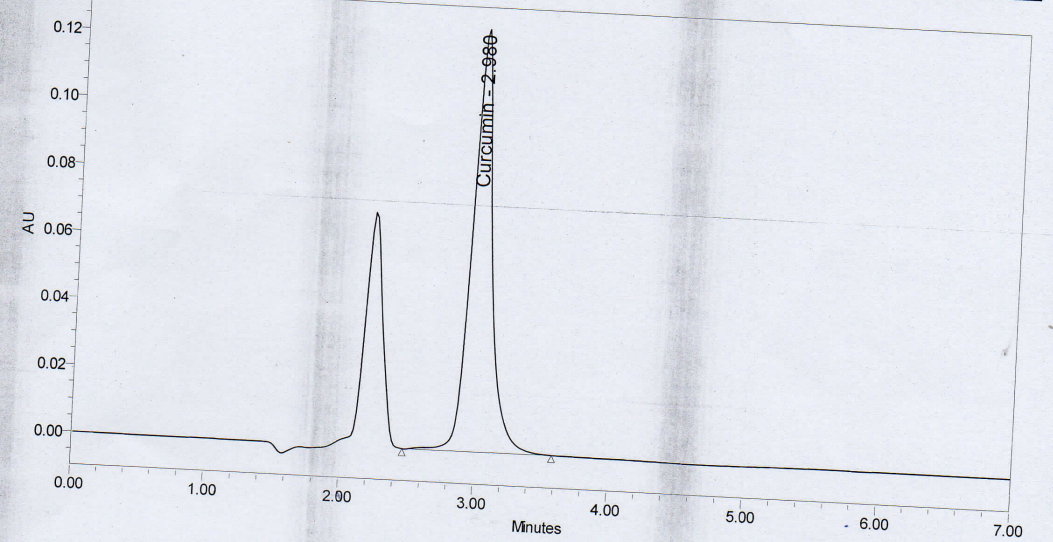
**

| **Peak Name** | **RT** | **Area** | **% Area** | **Height** |
| --- | --- | --- | --- | --- |
| Curcumin | 2.980 | 1328319 | 100 | 125624 |

**Curcumin standard 110%**

**
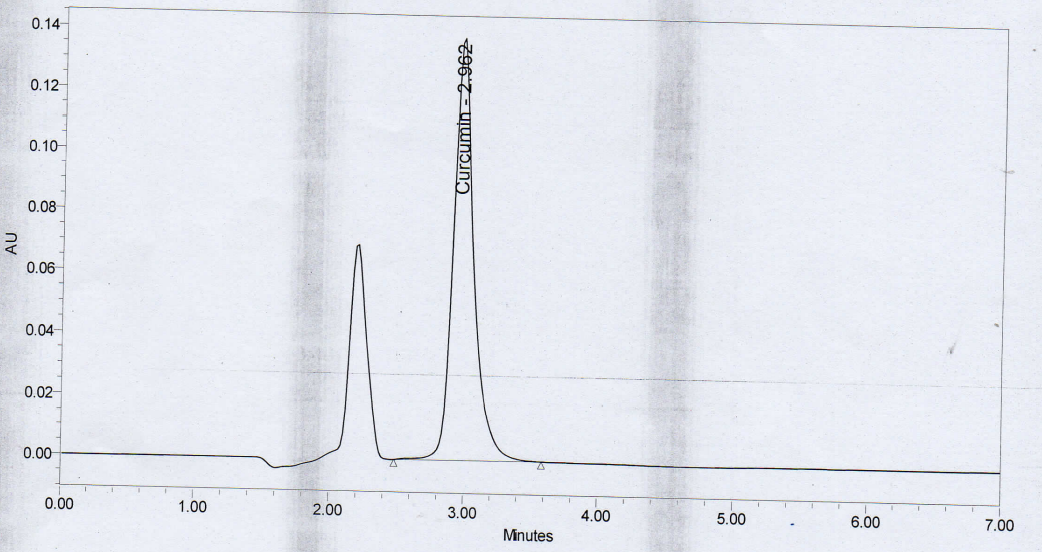
**

| **Peak Name** | **RT** | **Area** | **% Area** | **Height** |
| --- | --- | --- | --- | --- |
| Curcumin | 2.962 | 1459606 | 100 | 137809 |

**Curcumin standard 110%**

**
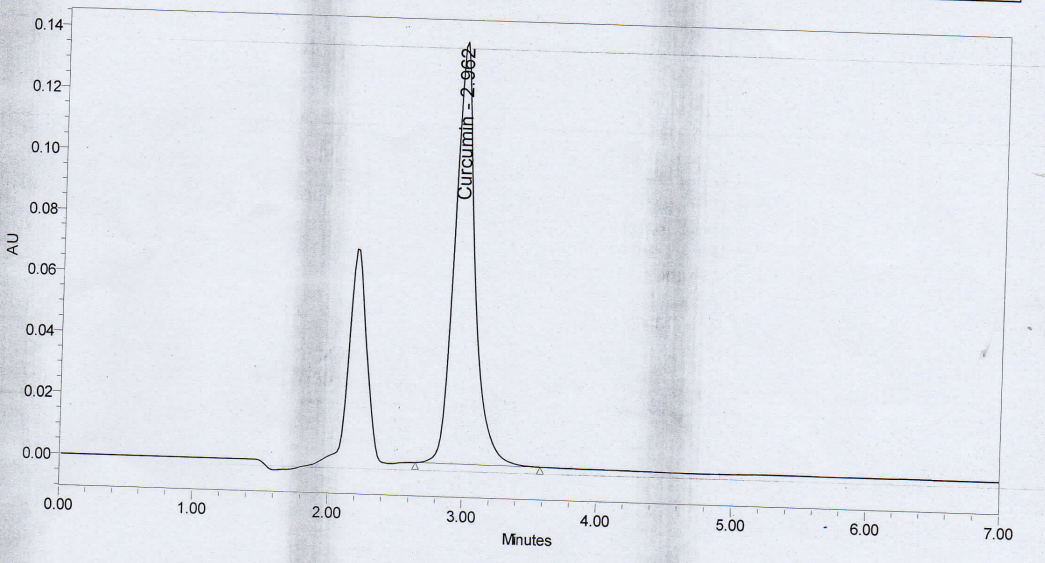
**

| **Peak Name** | **RT** | **Area** | **% Area** | **Height** |
| --- | --- | --- | --- | --- |
| Curcumin | 2.962 | 1448347 | 100 | 137895 |

**Curcumin standard 120%**

**
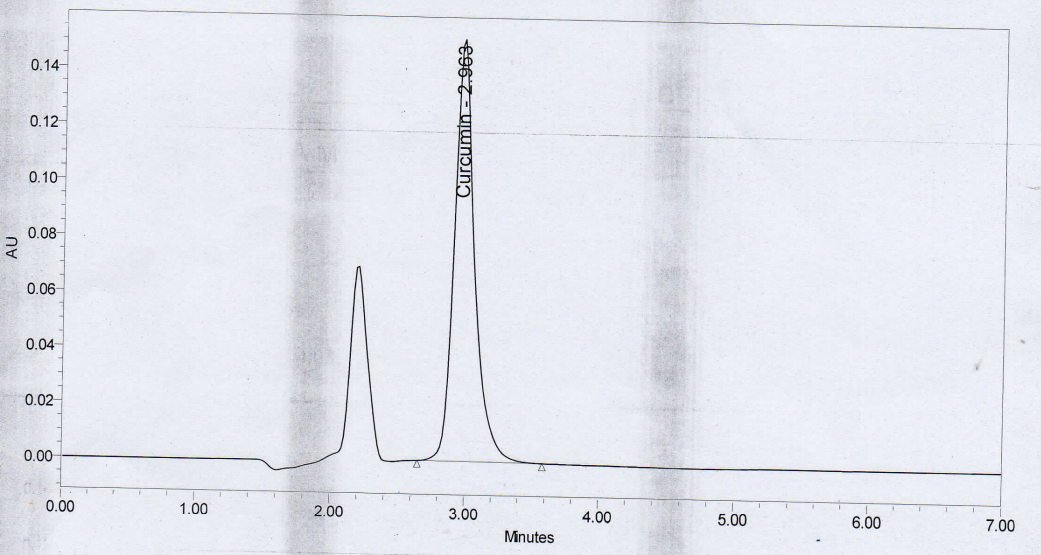
**

| **Peak Name** | **RT** | **Area** | **% Area** | **Height** |
| --- | --- | --- | --- | --- |
| Curcumin | 2.963 | 1583091 | 100 | 151199 |

**Curcumin standard 120%**

**
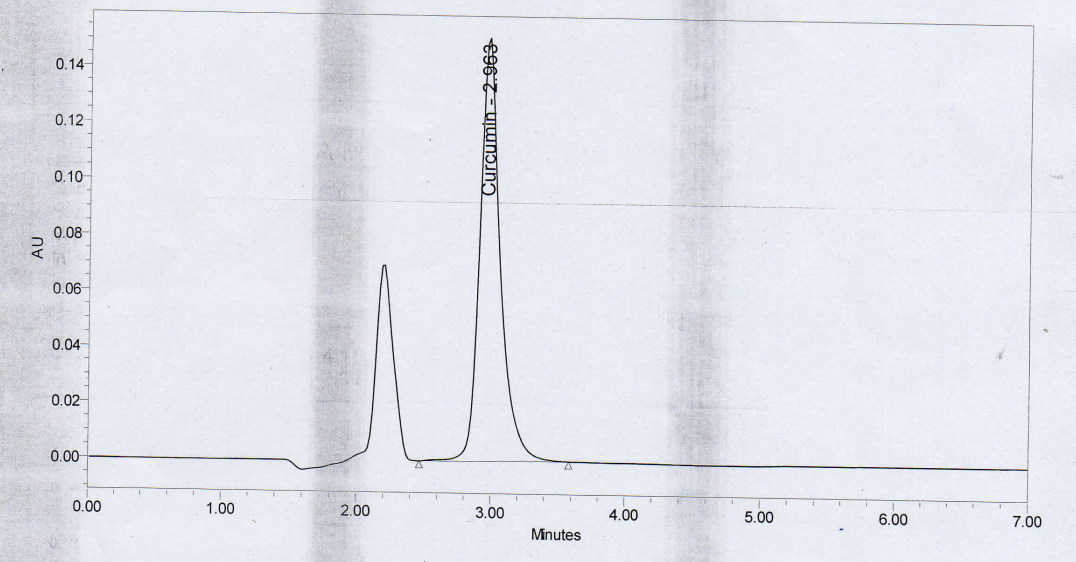
**

| **Peak Name** | **RT** | **Area** | **% Area** | **Height** |
| --- | --- | --- | --- | --- |
| Curcumin | 2.963 | 1600885 | 100 | 151270 |

**System precision**

| **Injections** | **Area** | **RT** |
| --- | --- | --- |
| 1 | 1340758 | 2.964 |
| 2 | 1368670 | 2.954 |
| 3 | 1334628 | 2.958 |
| 4 | 1320462 | 2.961 |
| 5 | 1312303 | 2.956 |
| Average | 1335364 | 2.959 |
| Standard deviation | 21753 | 0.00 |
| % RSD | 1.6. | 0.13 |
|  | NMT | NMT |
| % RSD limit | 2% | 2% |

**Curcumin standard 1**


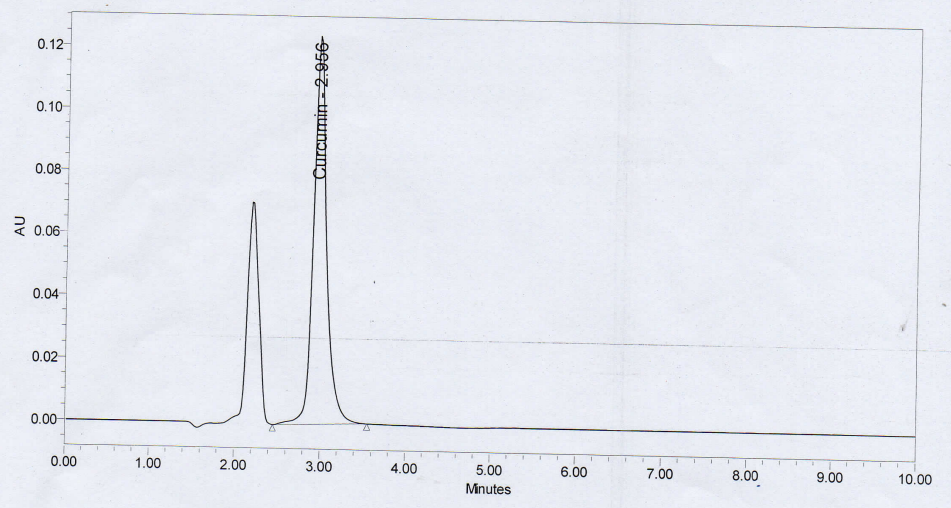


| **Peak Name** | **RT** | **Area** | **% Area** | **Height** |
| --- | --- | --- | --- | --- |
| Curcumin | 2.956 | 1312303 | 100 | 124586 |

**Curcumin standard 2**

**
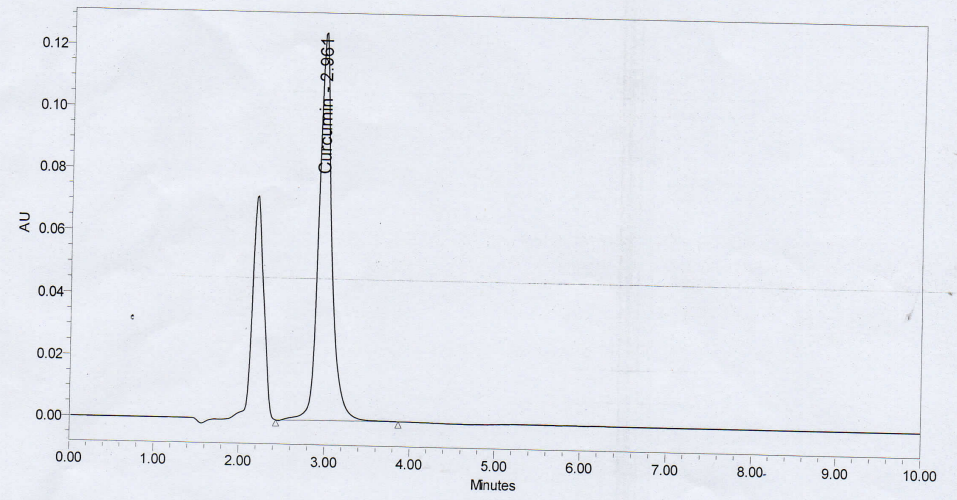
**

| **Peak Name** | **RT** | **Area** | **% Area** | **Height** |
| --- | --- | --- | --- | --- |
| Curcumin | 2.961 | 1320462 | 100 | 125415 |

**Curcumin standard 3**

**
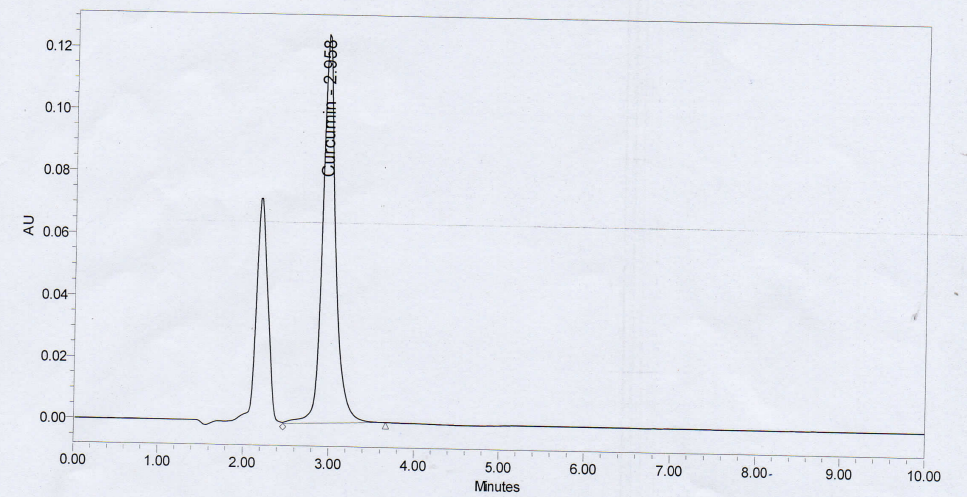
**

| **Peak Name** | **RT** | **Area** | **% Area** | **Height** |
| --- | --- | --- | --- | --- |
| Curcumin | 2.958 | 1334628 | 100 | 126209 |

**Curcumin standard 4**

**
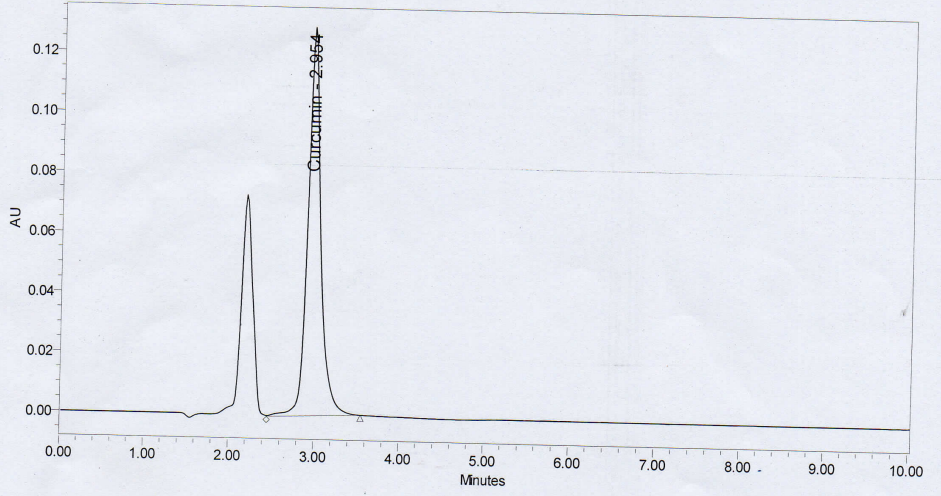
**

| **Peak Name** | **RT** | **Area** | **% Area** | **Height** |
| --- | --- | --- | --- | --- |
| Curcumin | 2.954 | 1368670 | 100 | 129093 |

**Curcumin standard 5**

**
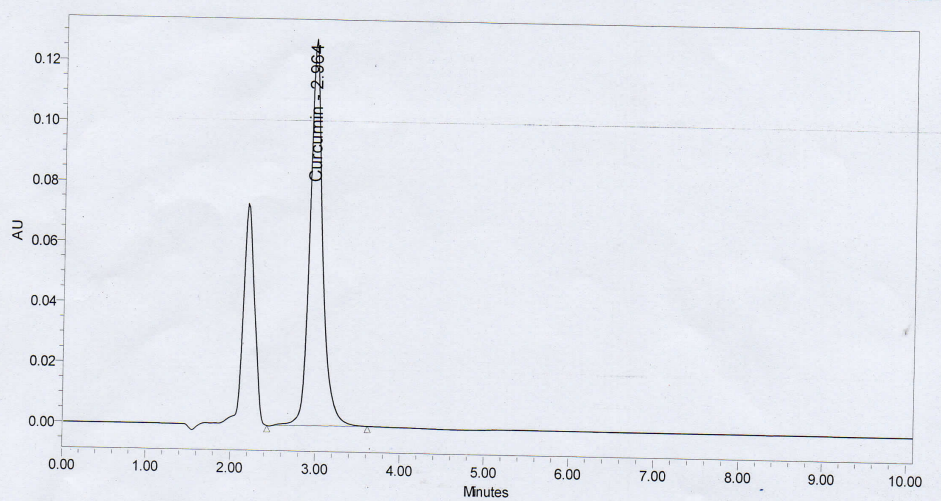
**

| **Peak Name** | **RT** | **Area** | **% Area** | **Height** |
| --- | --- | --- | --- | --- |
| Curcumin | 2.964 | 1340758 | 100 | 127636 |

**Results for the analysis of curcumin content in Mulmina Mango**

| **Test substance** | **Area** |
| --- | --- |
| Sample | 1923031 |
|  | 2023500 |
| Average | 1973261 |
| Standard | 2441092 |
|  | 2444331 |
| Average | 2442712 |

**The curcumin content of the sample was found to be 3.11mg/200ml**

**Curcumin Sample 1**


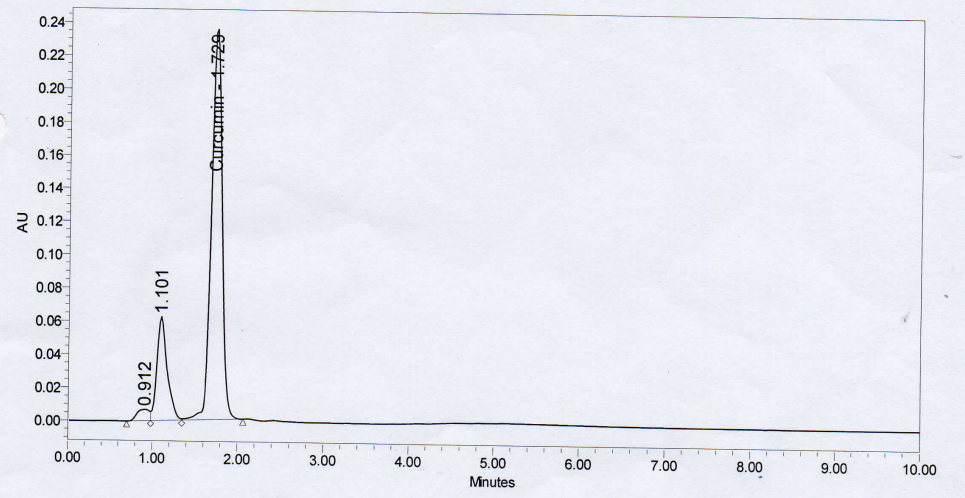


| **Peak Name** | **RT** | **Area** | **% Area** | **Height** |
| --- | --- | --- | --- | --- |
|  | 0.912 | 69601 | 2.66 | 6859 |
|  | 1.101 | 524833 | 20.05 | 62162 |
| Curcumin | 1.729 | 2023500 | 77.29 | 235388 |

**Curcumin Sample 2**

**
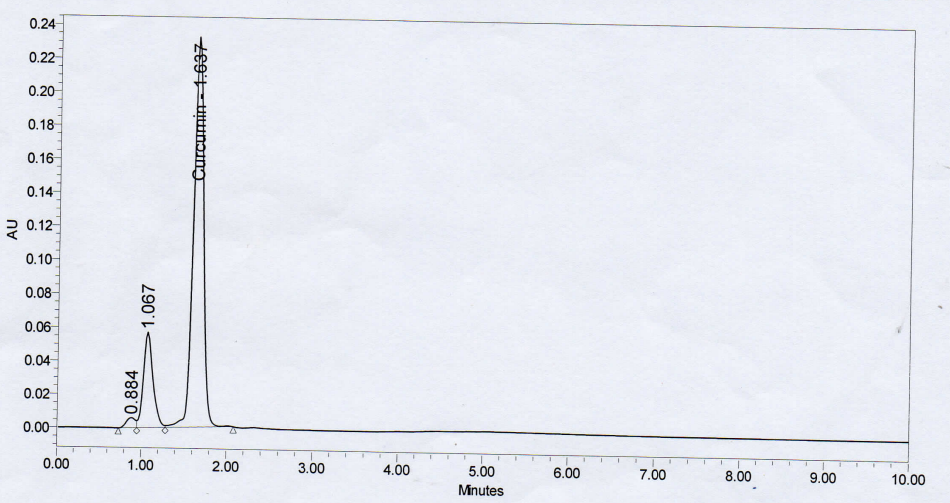
**

| **Peak Name** | **RT** | **Area** | **% Area** | **Height** |
| --- | --- | --- | --- | --- |
|  | 0.884 | 44317 | 1.84 | 5967 |
|  | 1.067 | 445544 | 18.47 | 56837 |
| Curcumin | 1.637 | 1923021 | 79.70 | 232177 |

**Curcumin standard 1**

**
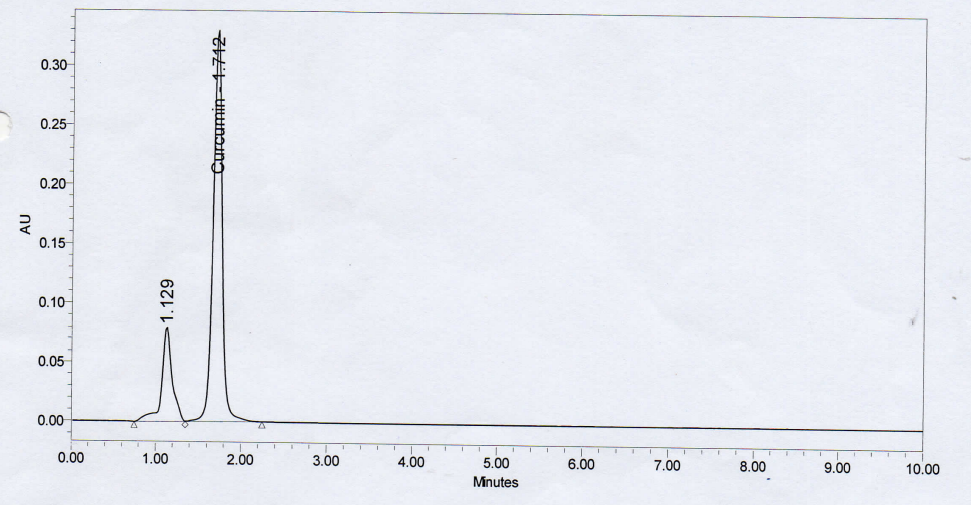
**

| **Peak Name** | **RT** | **Area** | **% Area** | **Height** |
| --- | --- | --- | --- | --- |
|  | 1.129 | 691439 | 22.07 | 80050 |
| Curcumin | 1.712 | 2441092 | 77.93 | 329882 |

**Curcumin standard 2**

**
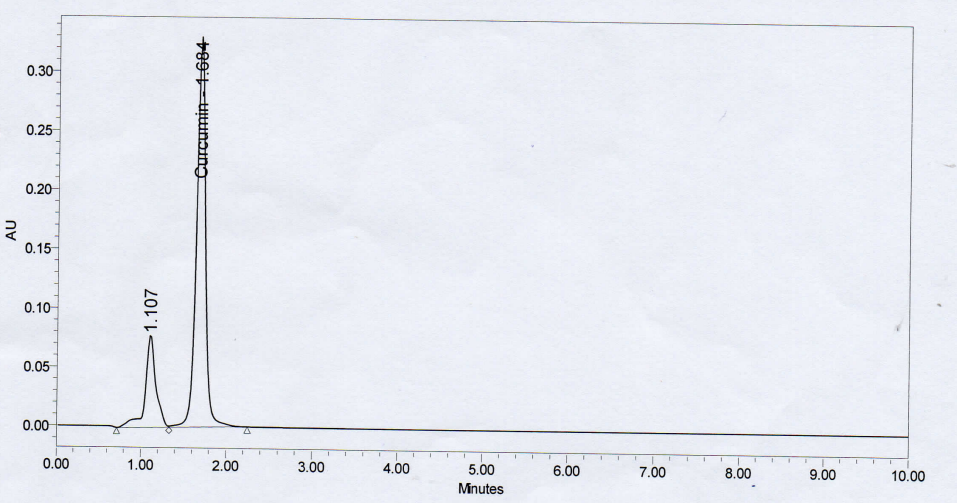
**

| **Peak Name** | **RT** | **Area** | **% Area** | **Height** |
| --- | --- | --- | --- | --- |
|  | 1.107 | 678713 | 21.03 | 78245 |
| Curcumin | 1.684 | 2444331 | 78.27 | 328544 |

1. **Open-field chambers**


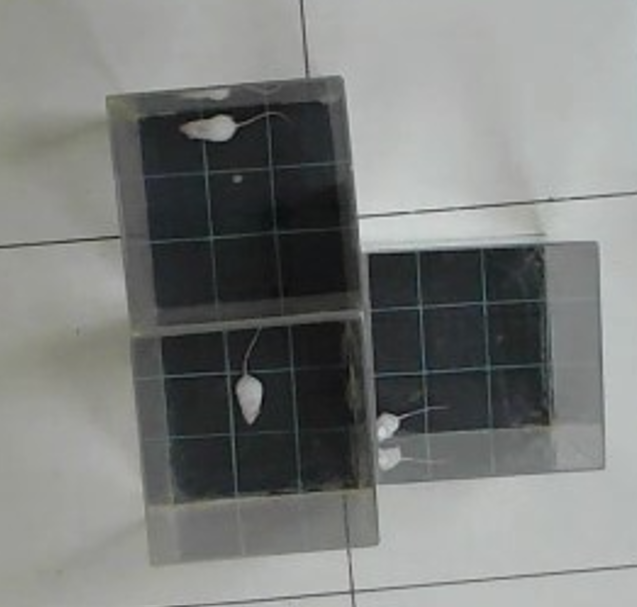


Figure 1: Glass chambers used in open field test
